# The infant–doctor relationship: an examination of infants’ distress reactions in the presence of a doctor

**DOI:** 10.1038/s41598-024-58677-5

**Published:** 2024-04-04

**Authors:** Motonobu Watanabe, Masaharu Kato, Yoshi-Taka Matsuda, Kosuke Taniguchi, Shoji Itakura

**Affiliations:** 1 Center for Baby Science, Doshisha University, 4-1-1 Kizugawadai, Kizugawa City, Kyoto 619-0225 Japan; 2 Department of Pediatrics, NHO Minami Kyoto Hospital, Joyo, Kyoto Japan; 3 Department of Child Studies, Shiraume Gakuen University, Kodaira, Tokyo Japan; 4 Department of Psychology, Kyoto University of Advanced Science, Ukyo-ku, Kyoto, Japan

**Keywords:** Paediatrics, Paediatric research, Medical ethics

## Abstract

Fear of doctors is a common source of distress among infants; however, the underlying sources of this distress are unknown. To investigate the doctor-infant relationship, the behaviors of 61 healthy infants (176–617 days old) were observed in a simulated examination room. Their behaviors and electrocardiograms were recorded. Two groups of infants were analyzed: those who cried and those who did not. When an experimenter dressed in the doctor’s attire entered the room, all 9 infants who were crying (14.8% of all infants) stopped crying, all infants gazed at the experimenter, and their mean heart rate (HR) decreased. After the auscultation started, 29.5% of all infants cried, and the HRs of infants who cried were higher than those of infants who did not cry. During the auscultation, 80.0% of infants who cried averted from the experimenter, while 34.4% of infants who did not cry. Within 5 s of gazing at the stethoscope, the number of infants who cried increased from 3 to 12, and their mean HR also increased. Our findings suggest that the fear of doctors is not due to the appearance of doctors but rather to specific actions performed by doctors, such as auscultation. Infants may regard a doctor’s appearance as a source of interest. Furthermore, a stethoscope is a possible trigger for infants’ crying. These behavioral observations suggest the potential for patient-centered care for infants.

## Introduction

Infants often cry, fuss, and refuse medical procedures when visiting a doctor^1–3^, and these behaviors represent medical distress, referred to as “fear of doctors.” A common belief is that infants are afraid of doctors; however, the mechanisms underlying these behaviors are unknown. Infant crying not only makes medical care difficult, but also may exacerbate cyanosis and other symptoms^4,5^. When providing medical care, pediatricians must develop a rapport with infants quickly to avoid distressing them^6,7^. Communication is the most common “procedure” in medicine^8^.

Forms of medical distress, such as those related to needle procedures, are often considered mild; however, for certain children, this distress is far from benign^9^. Moreover, anxiety-inducing experiences, such as being hospitalized and receiving medical care, can affect physical growth, personality, and emotional development^1^. These negative experiences in childhood may lead not only to medical fear but also to the avoidance of medical care as an adult^10,11^. Despite these problems, the medical care field often neglects to consider children's thoughts and emotions. Health care providers have a moral and ethical obligation to discuss health and illness with child patients, just as they do with adults^8^.

Recently, patient-centered care has played an increasing role in building partnerships between patients and healthcare professionals^12–16^. However, regarding pediatric patients, most studies have focused on family-centered care^17–19^. Few reports have examined patient-centered care in childhood, particularly in infancy. One reason for this lack of reports may be that infants have limited communication skills and cannot convey their specific wishes. An assessment is needed to determine whether family-centered care truly leads to positive experiences for children^20^. With respect to patient-centered care for infants, we propose that examining the factors underlying the fear of doctors is critical.

A meaningful approach is for pediatricians to elucidate the cause of infants’ crying during medical procedures. Many studies have used questionnaires to show that the visual perceptions of doctors reflect children’s comfort levels^6,21,22^. In addition, children’s self-reported distress is significantly associated with their attitudes toward healthcare^23^. However, little is known about how infants (at a preverbal age) feel about doctors or healthcare. Hyson observed young children of different ages during routine visits to a doctor’s office and described the effects of age and time period or situation on negative emotions^24^. However, certain problems exist with the methods used in Hyson’s study. One problem is that the conditions were uncontrolled because the observations occurred in a real-world medical setting. Another problem is that all the results were based solely on the judgment of one examiner’s observations every 30 s and lacked objective video data^24^. Infants may change their responses to physicians every second during an examination. Therefore, we need to expand upon previous research by including more detailed identifications of facial expressions and behaviors on a second-by-second basis. Moreover, the use of images alone is a limitation, and it is necessary to consider other indicators. Even when analyzing video recordings, coders are limited in their ability to understand infants' ever-changing facial expressions. In addition, it is assumed that there may be cases in which it is difficult to make judgments regarding the interpretation of facial expressions. Therefore, we measured HR as another indicator because electrocardiography (ECG) provides a physiological indicator that captures aspects of the emotional response to stimuli, such as activation of the autonomic nervous system. Heart rate has been considered to be an index of emotion. For example, a deceleration in HR is considered a measure of interest or attention, whereas an acceleration in HR is regarded as an indicator of fear^25–27^.

The current study investigated the doctor-infant relationship by focusing on infant reactions to determine a method for examining infants without provoking crying. The study was designed to analyze the differences between two groups: those who cried and those who did not. We obtained fine-grained distinctions by coding the video footage taken during a physical examination. The infants’ facial expressions and gazes recorded in the video were coded at 0.2 s intervals. Additionally, HR analyses were adopted to examine the transition of infants’ emotions as a method other than facial expression surveys. We hypothesized that the infants’ HRs would fluctuate with the scenes, even if their expression did not change. The questions addressed in this study were as follows:

1. Are infants afraid of doctors if a doctor appears and approaches them? We hypothesized that the infants’ crying could be attributed to the doctor’s approach and relative distance from the doctor. If infants are afraid of doctors, they may cry or avert their gaze from a doctor as soon as they see a doctor, and their HR will be accelerated.
2. What type of scene during a medical examination causes infants to start crying? We divided the consultation process into three scenes. Scene 1: An experimenter wearing a white coat enters the room after knocking on the door and approaches the infant (first contact scene). We investigated whether the medical distress of infants was due to the approach of doctors in Scene 1. On the other hand, if infants are interested in doctors, they may look at the experimenter without crying and their HR may decelerate. Scene 2: The experimenter sits down on a chair and interviews the infant’s mother (interview scene). We needed to ascertain whether the infants’ distress had been relieved or worsened during the interviews. We postulated that maternal engagement with the doctor would dampen alarms and infants would gaze at the experimenter with their HR decelerated in Scene 2. Scene 3: The experimenter takes a stethoscope out of his pocket and places it on the infant's chest (auscultation scene). We needed to explore whether infants cry due to medical practices such as auscultation. We also paid attention to the infants’ gaze and HR before and after auscultation.
3. What triggers infant crying? To further understand whether there were particular aspects of the scenes that triggered infant crying, we analyzed video data before and after infants cried. A more detailed analysis of the scene confirmed what factors caused the crying. We investigated what infants were watching and how their HR changed before crying in particular.
4. What individual factors are associated with infant distress? We investigated whether factors in an infant’s life and temperament were associated with fear of doctor. The infants’ temperament was measured using the Infant Behavior Questionnaire (IBQ)^28^. Rothbart et al. defined temperament as constitutionally based individual differences in reactivity and self-regulation^29^. Reactions to doctors may be related to infants' individual temperaments, especially their ability to self-regulate. The independent variables associated with the infants’ crying were assessed using a stepwise multivariate logistic regression model. We added the IBQ data to the results of a stepwise logistic regression analysis.

Here, to examine the fear of doctors in infancy, we reproduced the medical examination situation and examined the behaviors of infants who cried or did not cry.

## Results

### Changes in facial expressions during each scene

**Figure 1:**
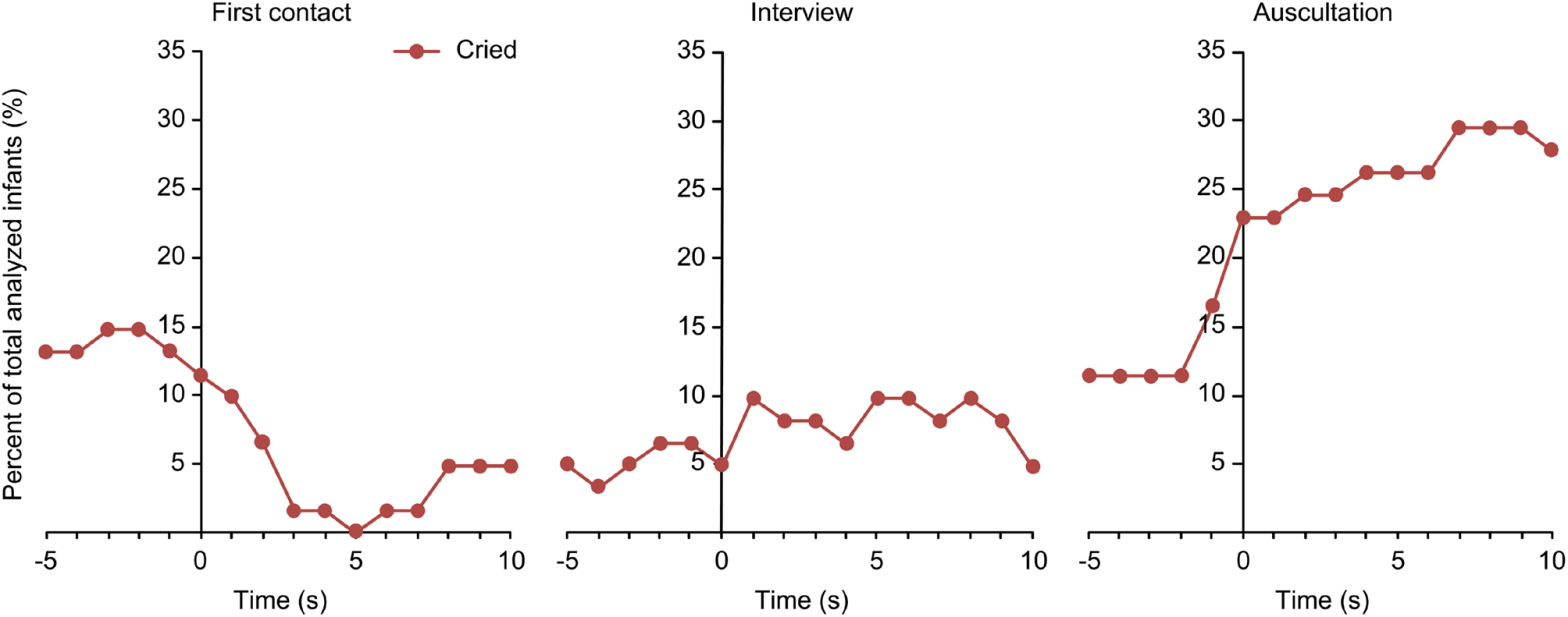
Changes in facial expressions during each scene. The time course of changes in the percentages of infants who cried in each scene is shown. The changes in facial expressions from 5 s before to 10 s after the event in each scene are shown. The 0-s time points are the times when the experimenter knocked on the door in the first contact scene, was seated in the interview scene, and placed a stethoscope on the infant’s chest in the auscultation scene.

The infants’ facial expressions were coded to examine whether the infants were afraid of the doctor and when they started crying. During the experiment, 28 infants cried, while 33 infants did not cry (Table 1). We analyzed how many infants cried from 5 s before to 10 s after the event in each scene (Fig. 1). In the first contact scene, nine infants (14.8% of all infants) cried 2 s before the experimenter entered the room. The infants stopped crying when the experimenter entered the room and approached them, and no infants cried at 5 s. The percentage of crying infants tended to be less than 10% in the interview scene. According to the analysis of infants’ facial expressions in the auscultation scene, the number of crying infants increased by approximately two-fold when the experimenter began the examination. Of the 28 infants who cried in this study, 21 cried from 5 s before to 10 s after auscultation. Moreover, 18 infants (29.5%) cried from 7 to 9 s after auscultation, the maximum value obtained in the experiment. This number decreased to 7 infants (11.7%) who cried for 5 s after the experimenter left the room (Supplementary Fig. S1).

**Table 1:** Baseline demographics and experimental characteristics.

No.^a^	Characteristic	Did not cry	Cried	Total
		(N = 33)	(N = 28)	(N = 61)
1	Postnatal age (SD)	361.3 (119.7)	395.5 (92.5)	377.0 (109.4)
2	Female sex, no. (%)	19 (57.6)	14 (50.0)	33 (54.1)
3	Gestational age at birth, weeks (SD)	38.9 (± 1.4)	38.6 (± 1.2)	38.8 (± 1.3)
4	Birthweight, g (SD)	3108.6 (± 484.4)	2880.4 (± 353.1)	3003.8 (± 444.0)
5	Number of household members, no. (%)			
	≤ 3	12 (36.4)	10 (35.7)	22 (36.1)
	4	14 (42.4)	13 (46.4)	27 (44.3)
	≥ 5	7 (21.2)	5 (17.9)	12 (19.7)
6	Siblings, no. (%)			
	0	18 (54.5)	11 (39.3)	29 (47.5)
	≥ 1	15 (45.5)	17 (60.7)	32 (52.5)
7	Attends nursery school/kindergarten, no. (%)	7 (21.2)	5 (17.9)	12 (19.7)
8	Shyness, no. (%)	28 (84.8)	19 (67.9)	47 (77.0)
9	Infant follows mother when she walks away, no. (%)	30 (90.9)	26 (92.9)	56 (91.8)
10	Distressed when mother leaves, no. (%)	26 (78.8)	21 (75.0)	47 (77.0)
11	Medical consultation frequency/month. (SD)	1.3 (± 0.8)	1.2 (± 0.6)	1.3 (± 0.8)
12	Days after the last vaccination, days (SD)	56.0 (± 56.4)	43.3 (± 55.4)	50.2 (± 56.3)
13	Infant's doctor wears a white coat, no. (%)	23 (69.7)	19 (67.9)	42 (68.9)
14	Experience of crying at doctor’s consultation, no. (%)	5 (15.2)	16 (57.1)	21 (34.4)
15	Breastfeeding, no. (%)	21 (63.6)	15 (53.6)	36 (59.0)
	IBQ score (SD)	2.86 (0.69)	2.86 (0.76)	2.86 (0.72)

Eight infants were not included in the analysis because of infant refusal (*n* = 5), because they were previously treated by the experimenter (*n* = 2), or because of a lack of recorded data due to technical error (*n* = 1). “Infant refusal” means that the infant was not sufficiently controlled to participate in the experiment.

^a^Information regarding the infant characteristics was obtained using the Infant Questionnaire for Research. The numbers from 1 to 15 correspond to the same numbers shown in the questionnaire (Table 3).

### Changes in HR during each scene

**Figure 2:**
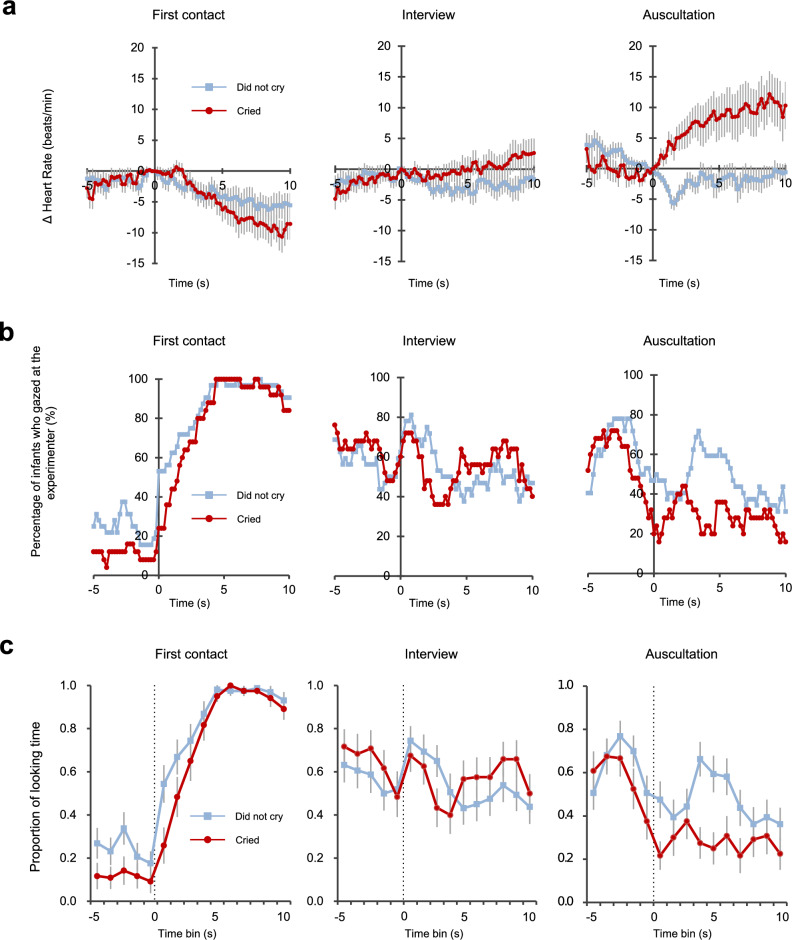
Changes in the infants’ HRs, gazes, and looking time during each scene. (**a**) The scene-related HR time courses in the two groups of infants (those who cried and those who did not cry) at each scene are shown. The changes in HR values refer to the HR relative to 0 s (delta HR). The results show the mean values for each group at each time point with the sample-by-sample intertrial SEM (vertical lines). (**b**) The percentages of infants who gazed at the experimenter in the two groups. Infants whose gazes were unable to be coded were excluded. In the scene without an experimenter, which occurred before he entered (the time before the 0-s time point), we counted the number of infants who gazed in the direction of the entrance door. (**c**) The proportion of the total looking time directed to the experimenter was averaged across all trials of the scenes for each successive 1-s time bin from 5 s prior to each scene until 10 s after the scene. Time 0 is indicated here with a vertical dashed line. The symbols with error bars plotted in each time bin represent the average data and standard errors.

We divided the infants into two groups (those who cried and those who did not cry) and compared the mean HRs between the groups during each scene (Fig. 2a). Due to missing data and noise, in the HR analysis we had to exclude data for 6 infants in the group that cried and 8 infants in the group that did not cry. Therefore, in the three scenes the numbers of included infants who cried and did not cry were 22 and 25, respectively. We conducted a series of hierarchical multiple regression analyses using generalized least squares methods with an autoregression structure (Supplementary Table S1).

For the first contact scene, we examined the goodness of fit of the statistics when autoregressive variables were included as covariates (i.e., HR ~ intercept vs. HR ~ intercept + autoregression) and found that the goodness of fit was significantly better when autoregressive variables were included as covariates (χ^2^(1) = 7680.44, p < 0.001). We then compared the goodness of fit of Model 0a, the intercept model with autoregressive covariance, with Model 1a, which adds two main effects (time course and two groups) to Model 0a (i.e., HR ~ intercept + autoregression vs. HR ~ time + group + autoregression). The results showed that the goodness of fit of Model 1a was significantly higher (χ^2^(2) = 14.29, p < 0.001). Furthermore, a comparison of the goodness of fit between Model 1a and Model 2a (i.e., HR ~ time + group + autoregression vs. HR ~ time * group + autoregression), in which the interaction effect was added to Model 1a, showed no significant change in goodness of fit (χ^2^(1) = 0.78, p = 0.376). Thus, an effect of time course or two-group interaction was not observed. Model 1a showed that the main effect of time course was significant and that HR decreased with time (beta = -0.40, p < 0.001). On the other hand, the effects in these two groups were not observed.

For the interview scene, the intercept model with autoregressive variables (Model 0a: HR ~ intercept + autoregression) was chosen because it showed a better fit than the intercept model without the variables (Model 0: HR ~ intercept; χ^2^(1) = 8235.13, p < 0.001). Model 1a (HR ~ time + group + autoregression) fit significantly better than Model 0a (χ^2^(2) = 9.28, p = 0.010), while the difference in the goodness of fit between Model 1a and Model 2a (HR ~ time * group + autoregression) was marginally significant (χ^2^(1) = 3.81, p = 0.051). Therefore, Model 1a was analyzed. The results indicated that the HRs of infants who cried were higher than those of infants who did not cry (beta = − 3.73, p = 0.014). The effect of the time course was marginally significant (beta = 0.22, p = 0.059).

During the auscultation scene, the intercept model with autoregressive variables (Model 0a: HR ~ intercept + autoregression) showed better goodness of fit than the intercept model (Model 0: HR ~ intercept; χ^2^(1) = 10,123.98, p < 0.001). The goodness of fit of Model 1a (HR ~ time + group + autoregression) was not significantly different from that of Model 0a (χ^2^(2) = 4.51, p = 0.105), while the goodness of fit of Model 2a (HR ~ time * group + autoregression) was significantly better than that of Model 0a (χ^2^(3) = 10.13, p = 0.018) and Model 1a (χ^2^(1) = 5.60, p = 0.018). Model 2a was chosen and indicated a significant effect of time course (beta = 0.51, p = 0.042) and interaction (beta = − 0.82, p = 0.017), while the effect on the two groups was not significant. The time course was centered at the median (2.5 s after the event) and at the value of the ¾ quartile deviation (6.25 s after the event) to analyze the simple slope. Both approaches indicated that the HRs of the infants who cried were significantly higher than those of the infants who did not cry after the event (betas < − 5.70, p < 0.028).

After the experimenter left the room (Supplementary Fig. S2a, Supplementary Table S1), the intercept model with autoregressive variables (Model 0a: HR ~ intercept + autoregression) showed a better fit than the intercept model without the variables (Model 0: HR ~ intercept; χ^2^(1) = 5132.25, p < 0.001). Model 1a (HR ~ time + group + autoregression) had a better fit than Model 0a (χ^2^(2) = 20.54, p < 0.001), while the goodness of fit of Model 2a (HR ~ time * group + autoregression) was not significantly different from that of Model 1a (χ^2^(1) = 1.87, p = 0.171). Model 1a showed a significant effect on the two groups (beta = 3.10, p = 0.030) and time course (beta = − 0.72, p < 0.001). The results indicated that the HR of the infants who did not cry was higher than that of the infants who cried and that the HR decreased with time.

**Figure 3:**
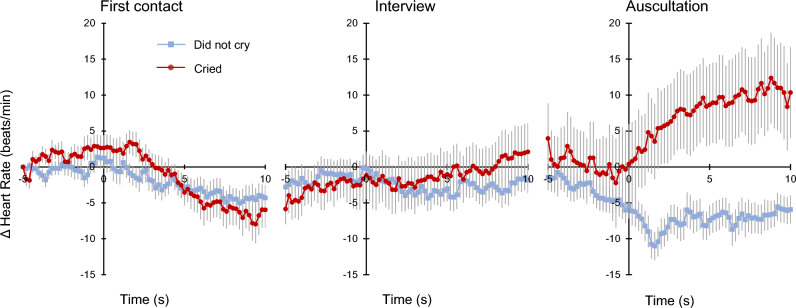
Comprehensive analysis with the measure before the event serving as the baseline. The baseline was defined as the HR 5 s before the first contact (5 s before the start of the experiment). Figure shows the HR time courses in the two groups. The changes in HR values in all scenes refer to the HR measured 5 s before the first contact scene. The symbols with error bars plotted in each time bin represent the average data and standard errors.

Moreover, when the relative HRs in all scenes were compared with the baseline values (i.e., HR at 5 s before the first contact scene) (Fig. 3), the first contact and the interview scenes showed no significant differences in HR between the two groups (Supplementary Table S1). For the auscultation scene, the intercept model with autoregressive variables was chosen (Model 0a: HR ~ intercept + autoregression) because it showed a better fit than the intercept model without the variables (Model 0: HR ~ intercept; χ^2^(1) = 12,343.50, p < 0.001). A comparison of the goodness of fit among the models revealed that Model 1a (HR ~ time + group + autoregression) was a marginally better fit than Model 0a (χ^2^(2) = 5.84, p = 0.054). Model 2a (HR ~ time * group + autoregression) was a significantly better fit than Model 0a (χ^2^(3) = 8.99, p = 0.029). Model 2a indicated that the HRs of infants who cried were higher than those of infants who did not cry (beta = − 9.00, p = 0.044). The effect of time course was not significant (beta = 0.46, p = 0.145), and the interaction was marginally significant (beta = − 0.76, p = 0.075).

### Changes in infants’ gazes and looking time during each scene

We coded the infants’ gazes at the experimenter in each scene (Fig. 2b). Three infants in the group who cried and one infant in the group who did not cry were excluded from the analysis because their gazes could not be coded from the video data. Therefore, the numbers of included infants who cried and did not cry were 25 and 32, respectively. During the time when the experimenter was not present (i.e., before he entered the room or after he left), we counted the number of infants who gazed in the direction of the entrance door.

In the first contact scene, fewer than 20% of the infants in each group gazed at the entrance door 1 s before the experimenter entered. However, infants in both groups began to gaze at the experimenter at 0 s, and all infants gazed at the experimenter within 4.6 s after he entered. For the looking time of the first contact scene, we examined whether the intercept model fit was changed by autoregressive variables (i.e., Model 0: looking time ~ intercept vs. Model 0a: looking time ~ intercept + autoregression) and found that the model with autoregression variables displayed a significantly better fit (χ^2^(1) = 978.05, p < 0.001; Fig. 2c, Supplementary Table S2). We compared the goodness of fit of Model 1a (looking time ~ time + group + autoregression) with Model 0a to investigate the effects of the two groups (infants who cried and those who did not cry) and time course and found that Model 1a displayed a significantly better fit (χ^2^(2) = 127.48, p < 0.001). Model 2a (looking time ~ time * group + autoregression), however, did not exhibit a significantly better fit than Model 1a (χ^2^(1) = 1.44, p = 0.230). Model 1a indicated that infants in both groups were more likely to look at the experimenter after he entered the room (beta = 0.06, p < 0.001), and infants who did not cry tended to look at the experimenter for a longer time (beta = 0.09, p = 0.067).

For the looking time of the interview scene, Model 0a (looking time ~ intercept + autoregression) showed a better goodness of fit than Model 0 (looking time ~ intercept; χ^2^(1) = 394.81, p < 0.001). Model 1a (looking time ~ time + group + autoregression) exhibited a marginally better fit than Model 0a (χ^2^(2) = 5.06, p = 0.080), and Model 2a (looking time ~ time * group + autoregression) was not a significantly better fit than either Model 0a or 1a (vs. Model 0a: χ^2^(3) = 5.08, p = 0.166; vs. Model 1a: χ^2^(1) = 0.01, p = 0.909). Therefore, neither the effects of the two groups nor the time course affected the viewing time.

No significant between-group differences were detected in the interview scene. However, during the auscultation scene, the percentage of infants who gazed at the experimenter in the group who cried was lower than that of the group who did not cry. At 3.6 s after auscultation started, 80.0% of infants who cried averted from the experimenter, whereas 34.4% of infants who did not cry. For looking time in the auscultation scene, Model 0a (looking time ~ intercept + autoregression) displayed a significantly better fit than Model 0 (looking time ~ intercept; χ^2^(1) = 432.04, p < 0.001). Model 1a (looking time ~ time + group + autoregression) fit significantly better than Model 0a (χ^2^(2) = 17.06, p < 0.001), while Model 2a (looking time ~ time * group + autoregression) did not fit significantly better. Model 1a indicated that infants who did not cry looked at the experimenter longer than infants who cried (beta = 0.12, p = 0.043), and the looking time at the experimenter decreased over time in both groups (beta = − 0.02, p < 0.001).

When the experimenter left, 88.0% of the infants who cried and 93.9% of the infants who did not cry gazed at the experimenter (Supplementary Fig. S2b). At 3.8 s after the experimenter left the room, 68.0% of the infants who cried gazed at the doorway through which the experimenter had exited, while the percentage of infants who did not cry decreased to 24.2%. For the looking time in the scene of leaving, Model 0a (looking time ~ intercept + autoregression) showed better goodness of fit than Model 0 (looking time ~ intercept; χ^2^(1) = 457.91, p < 0.001; Supplementary Fig. S2c). Model 1a (looking time ~ time + group + autoregression) showed a significantly better fit than Model 0a (χ^2^(2) = 39.48, p < 0.001), and Model 2a (looking time ~ time * group + autoregression) had a significantly better fit than Model 1a (χ^2^(1) = 5.04, p = 0.025). The regression analysis of Model 2a showed that the main effect of time course (beta = − 0.03, p = 0.009) and the interaction were significant (beta = − 0.03, p = 0.025). We examined the median (at the time of the event) and the value of the ¾ quartile deviation (2.5 s after the event) of the time course to test for a simple slope and found no difference between the two groups in the time spent looking at the experimenter up to the time of the event. However, the infants who cried were more likely to gaze at the doorway than those who did not cry after the experimenter left the room (beta = − 0.15, p = 0.051).

### Association with the stethoscope

**Figure 4:**
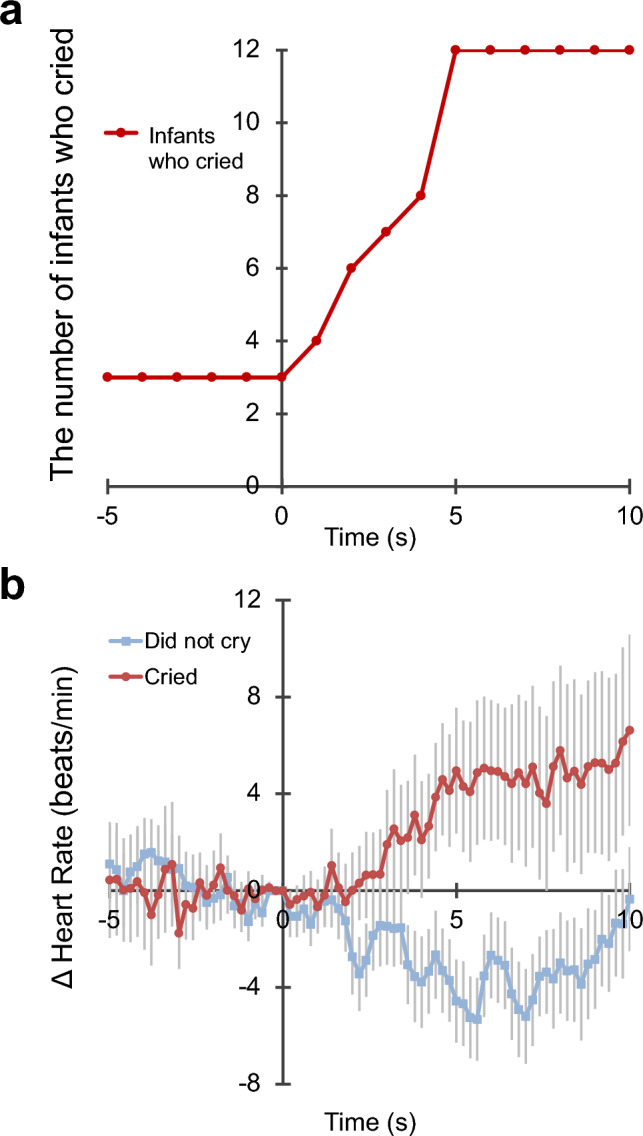
Association with the stethoscope. (**a**) The number of infants who cried before and after gazing at the stethoscope is shown. (**b**) The time course of the changes in HR (delta HR) before and after gazing at the stethoscope is shown. Infants who did not gaze at the stethoscope and whose HR data had noise or defects in each scene were excluded. The results show the mean values for each group at each time point with the sample-by-sample intertrial SEM (vertical lines).

We analyzed the behaviors of 28 infants before and after crying to determine which factors triggered their crying. Twenty-three (82.1%) of the infants who cried and 30 (90.9%) of the infants who did not cry began to watch the stethoscope before, during, or after auscultation. Within 5 s of gazing at the stethoscope, the number of infants who cried increased from 3 to 12 (Fig. 4a).

We examined whether the time course of HR before and after gazing at the stethoscope differed between the two groups (those who cried and those who did not cry) (Fig. 4b, Supplementary Table S3). The intercept model with autoregressive variables (Model 0a: HR ~ intercept + autoregression) showed better goodness of fit than the intercept model without the variables (Model 0: HR ~ intercept; χ^2^(1) = 8119.86, p < 0.001). Although no significant difference in goodness of fit was found between Model 0a and Model 1a (HR ~ time + group + autoregression; χ2(2) = 4.47, p = 0.107), Model 2a (HR ~ time * group + autoregression) had a significantly better fit than Model 0a (χ2(3) = 9.64, p = 0.022). Therefore, Model 2a was chosen as the optimal model. Model 2a indicated a significant effect of time course (beta = 0.43, p = 0.031) and interaction (beta = − 0.61, p = 0.022), while the effect of the two groups was not significant. To conduct the analysis of simple slopes, the time course was centered at the median (2.5 s after the event) and at the value of the ¾ quartile deviation (6.25 s after the event), both of which indicated that the HRs of the infants who cried were significantly higher than those of the infants who did not cry after gazing at the stethoscope (after 2.5 s: beta = − 3.57, p = 0.035; after 6.25 s: beta = − 5.84, p = 0.003). Thus, the HR of infants who cried was significantly higher than that of infants who did not cry as time elapsed.

### Logistic regression analysis

We used stepwise logistic regression analysis to determine crying triggers based on the infants’ profiles (Table 2). Previous experience crying at a doctor consultation was significantly associated with crying (OR, 15.09; p < 0.001). Low birth weight was also associated with crying; however, the association was not significant (OR, 1.00; p = 0.037). According to the results from the regression model, none of the other variables analyzed, including age and IBQ score, were significant.

**Table 2:** Comparison of the odds ratios for the characteristics of infants who cried and infants who did not cry.

Characteristic	Did not cry (n = 33)	Cried (n = 28)	OR	95% CI
Birth weight, g (SD)	3109 (± 484)	2880 (± 353)	1.00	0.997–1.000*
Siblings, no. (%)			1.90	0.80–4.67
0	18 (54.5)	11 (39.3)		
≥ 1	15 (45.5)	17 (60.7)		
Medical consultation frequency, /month (SD)	1.3 (± 0.8)	1.2 (± 0.6)	0.45	0.16–1.03
Days after the last vaccination, days (SD)	56.0 (± 56.4)	43.3 (± 55.4)	0.99	0.98–1.00
Experience of crying, no. (%)	5 (15.2)	16 (57.1)	15.09	3.75–82.20***

OR, odds ratio; CI, confidence interval.

*p < 0.05 and ***p < 0.001.

## Discussion

We examined the relationship between doctors and infants, focusing on infants’ reactions. In this study, four conclusions were drawn. (1) All of the infants gazed at an experimenter dressed in doctor’s attire without crying when he appeared and approached them. (2) Among the three scenes, most infants cried during the auscultation scene. (3) The number of infants who cried increased immediately after the experimenter used a stethoscope. (4) Infants’ crying was associated with a history of crying at doctor consultations.

The first goal was to show that infants were not afraid of the experimenter when he appeared and approached them during the first contact scene. The infants stopped crying at the moment they recognized the experimenter’s appearance. Similar to the infants who did not cry, the HRs of the infants who cried decreased, and they gazed at the experimenter after he entered the room. Most studies have focused on children’s perceptions of physicians’ attire^6,21,22^. Based on our results, the doctor’s appearance does not explain why the infants cried at the doctor’s office.

Second, of the 28 infants who cried, 21 infants cried during the auscultation scene, particularly immediately after the auscultation began. Moreover, these infants averted their gazes from the experimenter, and their HRs were faster than those of the infants who did not cry. We propose two possible explanations for this finding: infants may simply fear medical procedures, including auscultation, or infants may regard an experimenter as a doctor when he or she starts an examination. Based on these results, the fear elicited in infants by doctors is due to the doctor’s actions, such as performing examinations, and is not related to the doctor’s appearance.

Third, by performing a behavioral analysis before and after crying, we found that the stethoscope was a potential trigger of the infants’ crying behaviors. We did not test whether the appearance of a stethoscope would scare the infants; therefore, no definitive conclusion can be drawn. However, in a real doctor’s office, infants are unlikely to cry immediately upon seeing a stethoscope when it is not in use (e.g., when it is placed on the desk or hanging on a doctor’s shoulder). A more likely explanation might be that the doctor handling the stethoscope by the doctor or the doctor’s action with a stethoscope becomes scary for infants during the process of the examination.

Fourth, logistic regression analysis revealed that infants with a history of crying at doctor consultations tended to cry in the present study. This finding indicates that our study effectively reproduced the hospital environment. Previous reports suggest that the fear of doctors might be due to an infant’s past experiences at doctors’ offices^1,30,31^. The results of the above logistic regression indicated that to further elucidate infants’ fear of doctors, it is necessary to investigate infants’ past experiences with medical institutions. On the other hand, the results from the regression analysis indicated that no other factors, including infant age or IBQ score, were associated with infant crying. To assess the possibility that fear of doctors appears in infants of different ages, infants across a wide age range (176–617 days of age) were analyzed. However, the present experiment did not show that the infants’ ages were associated with their crying. A study that included older children suggested effects of age on negative emotions during pediatric examinations^24^. Future research should examine older infants or toddlers to establish the effect of children’s age on responses to doctors. Fear from the IBQ domain, which assesses distress in response to a novel social or physical object, was not a significant factor in the current regression model. If all domains were also examined, the relationship with temperament might have become clearer. Alternatively, using the IBQ-Revised (IBQ-R), which has been used as a measure of infant self-regulation, could have shown individual differences^32^.

Our results revealed other between-group differences, except in the auscultation scene. First, the infants who cried were more likely to gaze at the doorway than those who did not cry after the experimenter left the room. The infants who cried tended to maintain their attention on the experimenter even if the experimenter was not visible, whereas the infants who did not cry were likely to lose interest in the experimenter as soon as he left the room. In addition, the HR of infants who cried was significantly lower than that of infants who did not cry after the experimenter left the room. Thus, the experimenter had a greater effect on the emotions of the infants who cried, suggesting that the fear of doctors had already formed in the memories of the infants who cried. This research also has the potential to explore aspects of infant self-regulation. Analysis of the gaze and HR of the group that cried after leaving suggested a possible response during the “recovery” phase after stressor removal. Infants who cried may not have fully recovered from the stress even after the experimenter left, perhaps because they were not able to self-regulate completely. On the other hand, infants who did not cry may have been better able to regulate their behavior, or perhaps they did not perceive the experimenter as a stressor.

Second, significant differences were observed in the results of the HR analysis during the interview scene: the HRs of the infants who cried were higher than those of the infants who did not cry. Although further research is needed, maternal engagement with doctors may influence the heart rate of infants during interviews. Unlike in the auscultation scene, simultaneous differences in the HR and the looking time results were not observed in the interview scene. These results suggest that gaze is different from HR as an indicator and might identify differences that are not present in HR values. The measurement of infants’ gazes may provide additional information on other factors, such as their interests, cognitions, and emotions^33–36^, which is essential for patient-centered care^37–40^.

This research highlighted that a doctor’s examination can act as a mild stressor for infants. These findings are consistent with the literature on infant development, especially as they relate to how infants self-regulate^32,41,42^. Self-regulatory processes can be observed on multiple levels, including the physiological, attentional, emotional, cognitive, and interpersonal domains of functioning^29,43–45^. The Face-to-Face Still-Face Paradigm suggested a relationship between heart rate and emotional reactivity to a stressor^46^. Overcoming the fear of doctors can be thought of as the process of developing self-regulatory abilities through various maturation processes, including the autonomic nervous system.

This study investigated infants’ responses to a doctor to shift the perspective of infant medical care from family-centered to patient-centered care. Medical care requires both family-centered and patient-centered care; however, little is known about patient-centered care in infancy because infants do not have verbal skills and cannot convey their specific wants, unlike their parents. Our approach aims to address the challenge of investigating less-known patient-centered care for infants. Our findings have implications for pediatric healthcare and clinical practice, as follows: (1) an infant’s first impression of a doctor is favorable, (2) medical procedures may cause fear in infants, (3) opportunities to build relationships with infants occur during the time before the medical examination, and (4) doctors should avoid showing infants medical instruments such as stethoscopes before they are comfortable with examinations. For example, it might be worth considering auscultating an infant’s back at the beginning so that the infant does not pay attention to the stethoscope. If these proposed recommendations are considered for routine pediatric care, patient-centered care for infants may become more practical for social implementation.

Our study has several limitations. First, our research replicated the first visit to an unfamiliar doctor. Infants exhibit different behaviors when they consult with their own physicians, and reports indicate that past medical experiences influence infant behaviors^47,48^. Second, our results may not be widely available in a sufficiently timely manner to achieve patient-centered care for infants soon. However, we documented one way of approaching such care and accumulated evidence by observing infant behaviors rather than by testing interventions. Patient-oriented research must be based on both patient observations and heterogeneity studies rather than the identification of the best intervention for each individual patient^39^. Furthermore, the age range was limited in our research, and it is necessary to expand the scope to include older patients. Future studies should investigate more and broader samples by age to examine developmental changes. Additionally, we believe that there are potential confounding factors and alternative explanations that could not be shown in our exploration. For example, this study could be extended by considering measurements of infant and maternal HR variability, aspects of attachment, maternal mental health, a detailed assessment of the marital relationship, parental attitudes, and medical experiences. It is essential to address these limitations and consider them in future research efforts.

## Conclusions

The aim of the present study was to examine infant-doctor relationships, focusing on infants’ reactions during examinations. Based on our findings, the fear of doctors among infants is due to specific actions, such as auscultation, performed by a doctor. Another major finding was that infants are not afraid of the doctor’s appearance but rather show interest in the doctor. Furthermore, we found that a stethoscope is a possible trigger for infants’ crying. These findings provide insights that are applicable to addressing infants’ fear of doctors or healthcare. Beyond family-centered care, the present study advances the establishment of patient-centered care for infants. Based on behavioral observations of infants, this study suggests the potential for infant-centered care.

## Methods

### Participants

From December 13, 2011, to July 19, 2016, 69 healthy full-term infants were recruited through various methods, including announcements in local magazines, the internet, and participant referrals. Among the infants who participated, 61 (33 females [54.1%]; mean age [standard deviation: SD], 377.0 [110.3] days) were included in the analysis. Infants with a wide age range (aged 176–617 days) were analyzed to assess the possibility that fear of doctors appeared in infants of various ages. Eight infants were examined and then excluded for one of the following reasons: the infant was not sufficiently controllable to participate in the experiment (infant refusal) (n = 5), the infant had previously been seen by the experimenter as a patient (not seen for the first time) (n = 2), and data were not recorded due to a technical error (n = 1). Table 1 lists the demographic characteristics of the participants according to the Infant Questionnaire for Research (see Table 3). We classified the infants into two groups (those who cried during the experiment and those who did not cry).

**Table 3:** Infant questionnaire for research.

1.	Age of baby		Days
2.	Sex		
3.	Gestational age at birth		Weeks
4.	Birth weight		Grams
5.	How many family members does your baby have?^57,58^		
6.	Does your baby have any brothers or sisters?^58,59^	Yes	No
7.	Does your baby attend a kindergarten or a nursery school?^60,61^	Yes	No
8.	Is your baby shy or afraid of unfamiliar adults?^62^	Yes	No
9.	When you walk away, does your baby follow you?^49–51^	Yes	No
10.	When you leave your baby alone, does she or he cry?^49–51^	Yes	No
11.	When was your baby’s last vaccination?^9,31^		
12.	How often does your baby visit a doctor?^1,3,31^		Months
13.	Does your baby’s doctor usually wear a white coat?^63^	Yes	No
14.	Has your baby ever cried while being examined by a doctor?^30^	Yes	No
15.	Do you still breastfeed your baby?^64^	Yes	No

### Ethics approval

Written informed consent was obtained from the infants’ parents prior to their participation. The study was conducted in accordance with the principles of the Declaration of Helsinki, and the study design was approved by the ethics committee of Doshisha University (#1354-1). All experiments were completed within 5 min to minimize the burden on the infants. Furthermore, we prioritized the infants’ condition and stopped the survey soon after the infants expressed being unwell or refused to participate.

### Experimenter and procedure

Our experimenter was a pediatrician who provided daily medical care in a hospital near the laboratory. All participants met him for the first time during the study because we aimed to observe the infants’ behaviors while interacting with a new doctor, thus excluding biases associated with previous consultations. In accordance with standard attire, the doctor wore a white coat and had a stethoscope in his pocket.

**Figure 5:**
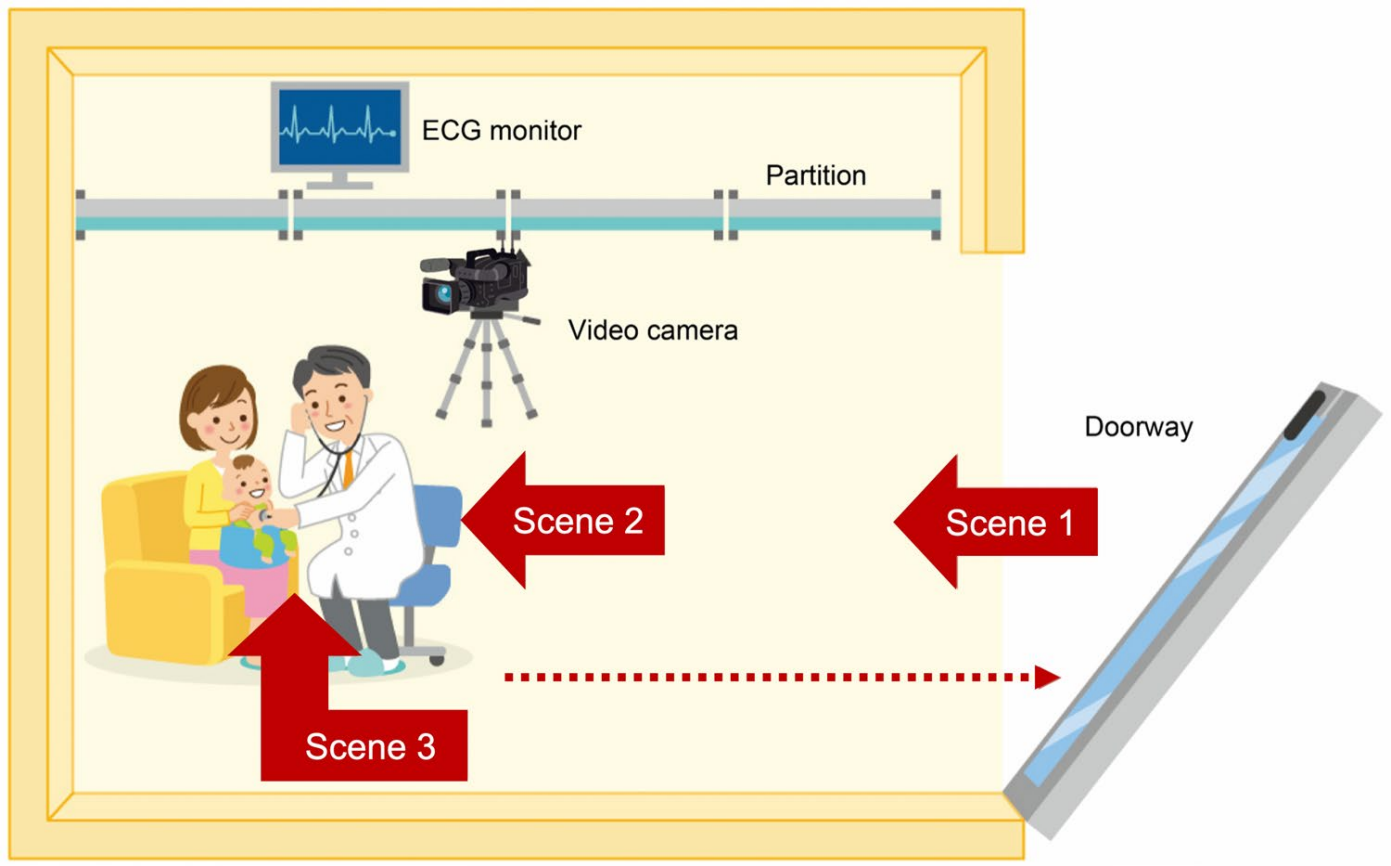
Experimental procedure in a simulated consultation room. A mother and an infant were introduced to a simulated consultation room, and they waited there for the doctor to enter. The following experimental procedure was used: Scene 1: An experimenter wearing a white coat enters the room after knocking on the door and approaches the infant (first contact scene). Scene 2: The experimenter sits down on a chair and interviews the infant’s mother (interview scene). Scene 3: The experimenter takes a stethoscope out of his pocket and places it on the infant's chest (auscultation scene). After the three scenes, the experimenter leaves the room (dotted arrow). An ECG recorder was placed behind the partitions. The digital video camera was covered with a black cloth such that the infant did not pay attention to it, but this figure does not show that process.

We planned to observe daily medical examinations of the infants. Thus, the experiments were conducted in a simulated consultation room in our laboratory. We used partitions and arranged the seats to resemble an examination room as much as possible (Fig. 5). In this experiment, we intended to confirm whether the fear of doctors was attributed to the doctor’s approach and the relative distance from the doctor. Therefore, a laboratory setup was devised to observe the extent to which infant behavior changed under the conditions of alarm introduced by the doctor’s arrival and approach^49–51^. The mother and infant were brought to the simulated consultation room, where they waited for the doctor to enter. The mother sat on a chair against the back wall, and her infant sat in a floor seat (Bumbo®, Bumbo International Trust, Pretoria, South Africa) on her lap. We used a Bumbo seat because we needed to exclude the effects of maternal touch and contact^52^. We recorded the infants with a digital video camera (Panasonic AG-DVX1008).

The experiment was constructed in three scenes (Fig. 5).

Scene 1: An experimenter wearing a white coat entered the room after knocking on the door and approached the infant (first contact). The moment of knocking was defined as the 0-s time point in Scene 1.

Scene 2: The experimenter sat down on a chair and interviewed the infant’s mother (interview). The moment of being seated was the 0-s time point in Scene 2.

Scene 3: The experimenter removed a stethoscope from his pocket and placed it on the infant’s chest (auscultation). The time of stethoscope placement was defined as the 0-s time point in Scene 3.

The observation period from 5 s before the scene to 10 s after the scene was considered to investigate infants’ responses in each scene. The 0-s time point indicated the start of the scene, and the observation started 5 s before the 0-s time point. We considered this time frame because in our review of initial data, we observed that infants may change their facial expressions every second once the doctor appears. After the three scenes, the experimenter left the room, and the experiment was complete. We also observed the infants’ behaviors before and after the experimenter left the room to determine the subsequent effects on the infants.

### ECG measurements

ECG data were used as physiological indicators. We attached an ECG sensor (PolyTele® STS-1C, Nihonsanteku Co., Ltd., Osaka, Japan) to the infant’s chest and measured the infant’s HR to investigate the effect on the autonomic nervous system. During each experimental period, ECGs were recorded and displayed using Audacity® software version 1.3 or 2.0. The sampling rate was set to 8000 samples/s, and no filtering was applied. Using the elapsed time from one heart contraction to the next (RR interval), the HR was calculated in beats per minute (bpm) with our in-house program in MATLAB® between consecutive RR interval samples. HR artifacts were confirmed and removed by MATLAB and visual inspection. If artifacts could not be removed, the data were removed from the analysis. Due to missing data and noise, we had to exclude data for 6 participants in the group that cried and 8 participants in the group that did not cry in the HR analysis.

### Video coding

The main outcome measures included the infants’ facial expressions and gazes as well as changes in their HRs. Two coders who were unaware of the study hypotheses identified the infants’ facial expressions and gazes. The facial expressions were coded as negative or not negative. Our criteria for defining negative facial expressions in infants were based on those described by Sullivan and Lewis^53^ or Ikeda and Itakura^54^. The criteria included lowering of the brows, tight squeezing of the eye orbital muscles, and lateral stretching of the mouth^55^. We defined vocalization with a negative facial expression as crying. The infants’ gazes were coded as looking at the experimenter’s face or looking elsewhere. A fixation time of more than 0.2 s shown on the video image was considered the looking time. The coding reliability was calculated for all samples using Cohen’s kappa coefficient^56^. The percentages of intercoder agreement were 94.6% for the infants’ facial expressions and 87.7% for their gazes. Three infants in the group that cried and one infant in the group that did not cry were not included in the results because these cases could not be coded.

### Infant questionnaire

We developed the Infant Questionnaire for Research, which consists of 15 questions, to collect data on infants’ background and temperament via mothers’ reports (Table 3). We investigated whether factors in an infant’s life, the mother-infant relationship, and the infant’s temperament were associated with fear of doctors. The variables included in the score were age, sex, gestational age, birth weight, family structure, the number of siblings, the level of shyness, the number of days since the last vaccination, medical consultation frequency, whether the infant attended a nursery school or cried at a doctor’s consultation, whether the doctor wore a white coat, and whether the infant followed his or her mother, cried when his or her mother left, or was breastfed^1,3,9,30,31,49–51,57–64^.

We also measured the infants’ temperaments using the Infant Behavior Questionnaire (IBQ)^28^. Although the IBQ is designed for infants aged 3–12 months, we used the IBQ items for all participants to directly compare their results using the same scale. The IBQ assesses six domains of infant temperament: fear, distress related to limitations, smiling and laughter, soothability, duration of orienting, and activity level. From six domains of the IBQ, we measured fear on a scale consisting of 16 items that assess distress in response to a novel, social, or physical object, such as a stranger. The IBQ score was included in the logistic regression analysis to determine the crying trigger based on the infant profile.

### Data analyses

We classified the infants into two groups (those who cried during the experiment and those who did not cry), which served as a dependent variable, to examine the differences between infants who cried and those who did not cry. We analyzed the infants’ behavior by comparing the reactions before and after the stimulus in each scene. In addition to facial expressions, we examined changes in the following measures to determine which scene induced distress: HR, the percentage of time infants spent gazing at the experimenter, and the time spent looking before and after the event in each scene. Throughout all the scenes, we analyzed the infants and paid particular attention to the moments before and after they cried.

Analyses of HR: After the selection of HR trials 5 s prior to and 10 s after each scene, averaging was performed across each scene. The results showed scene-related HR time courses with a sample-by-sample intertrial standard error of the mean (SEM). For statistical analyses, we extracted the relative HR values every 0.2 s for a time interval of 15 s in each scene^65,66^. The changes in HR values refer to the relative HR from the 0-s time point. In addition, we compared HRs across the different scenes for each group. The baseline was defined as the HR 5 s before the first contact (5 s before the start of the experiment). The changes in HR values across the different scenes were also analyzed by referring to the relative HR from the baseline. We conducted a series of hierarchical multiple regression analyses using generalized least squares methods with an autoregression structure in each scene and the nlme package in R version 4.1.0 (https://www.r-project.org) to examine whether the HR time course differed between the two groups (i.e., infants who cried and those who did not cry). The model included HR as the dependent variable. The time course of a scene (at intervals of 0.2 s; centered at the event occurrence) was set as the continuous independent variable, and whether the infants cried or did not cry was set as the dummy independent variable. The time course of the scene and infant ID were set as the covariates for the time and grouping factors, respectively. A p value of < 0.05 was considered significant for all analyses. We compared the goodness of fit (AIC) between models using the likelihood ratio test to analyze the effect of independent variables. The models to be compared are described below. First, we examined whether the goodness of fit differed depending on whether the residuals included or did not include autoregression in the intercept model only to control for the effect of the time course. If the fit of a model including the autoregressive covariate was high, it was set as Model 0a. Otherwise, the autoregressive covariate was not included (Model 0). Model 1 (or Model 1a) was added to Model 0 (or Model 0a) to include the main effects of time course and whether the infant group cried or did not cry, and Model 2 (or Model 2a) was added to Model 1 (or Model 1a) to include the effect of the interaction.

We analyzed the changes in the infants’ gazes from 5 s before the scene to 10 s after each scene. Analyses of the percentages of the infants who gazed at the experimenter: The percentages of infants in the two groups that gazed at the experimenter in each scene were calculated every 0.2 s and were displayed as a time series. Analyses of looking time: The proportion of the total looking time directed to the experimenter was averaged across all trials of the scenes for each successive 1-s time bin from 5 s prior to 10 s after the event of each scene^67^. For looking time, a linear model with an autoregression structure fit was also run. The analysis procedure was similar to that for HR except that the dependent variable was set as the looking time and the time course was set at intervals of 1 s.

We analyzed the contributions of the predictor variables to determine the effects of individual differences. The independent variables associated with the infants’ crying were assessed using a stepwise multivariate logistic regression model with R. The data were obtained using the Infant Questionnaire for Research and the IBQ (Table 1). We performed a logistic regression analysis to estimate the odds ratios and 95% confidence intervals (CIs) for the risk of crying. A p value < 0.05 was considered significant for all analyses.

### Consent to participate

Written informed consent was obtained from the infants’ parents prior to their participation.

### Consent for publication

Verbal informed consent was obtained from all individual participants to publish nonidentifying information collected during the experiment.

### Supplementary Information

Supplementary Information 1.

Supplementary Figures.

Supplementary Tables.

## Data Availability

All data generated or analyzed during this study were included in this published article and its supplementary information files (Supplementary Data 1).
