# Supplementary material for: The infant–doctor relationship: an examination of infants’ distress reactions in the presence of a doctor
Source: Sci Rep. 2024 Apr 4;14:7968. doi: 10.1038/s41598-024-58677-5 (PMC10994921; doi:10.1038/s41598-024-58677-5)
Supplement: Supplementary file 1 — Supplementary Information 1. [file 41598_2024_58677_MOESM1_ESM.pdf]

	**Supplementary Data 1 (S1 Data)**
article title	**The Infant-Doctor Relationship: An Examination of Infants' Distress Reactions in the Presence of a Doctor**
journal name	**Scientific Reports**
author names	**Motonobu Watanabe*, Masaharu Kato, Yoshi-Taka Matsuda, Kosuke Taniguchi, Shoji Itakura**
affiliation and e-mail address of the corresponding author.	***Center for Baby Science, Doshisha University, 4-1-1 Kizugawadai, Kizugawa-city, Kyoto 619-0226, Japan<br>E-mail: mowatana@mail.doshisha.ac.jp**

Raw data used for analyses

Explanation for data files:

Sheet 1. Charasteristics This sheet summarizes the raw data of the characteristics for participants.

Column	Explanation	Units / Meaning of codes	
age	Age of baby	days	
sex	Gender	0 = Female	1 = Male
Gestational age	Gestational age at birth	weeks	
Birth Weight		gram	
family	Number of household members		
sibling		0 = None	1 = One or more siblings
nursery	nursery/kindergarten pupil	0 = No	1 = Yes
shyness	Infant is shy or afraid of an unfamiliar adult	0 = No	1 = Yes
follow	Infant follows mother when she walks away	0 = No	1 = Yes
wo_mom	Distressed when mother leaves	0 = No	1 = Yes
Consultation frequency	Medical consultation frequency	/month	
Injection	Days after the last vaccination	days	
white coat	Infant's doctor wears a white coat	0 = No	1 = Yes
Experience of crying	Experience of crying at doctor's consultation	0 = No	1 = Yes
breast_feed	Mother still breastfeeds her infant	0 = No	1 = Yes
Cry	Crying during the experiment	0 = No	1 = Yes

Sheet 2. Infants Who Cried in Each Scene.

Columns	Explanation	Notes / Meaning of codes
Time (s)	Time course before and after the event in each Scene	1 = Cried<br>Blank = Did not cry<br>Yellow cell = Lack of data due to technical error

Sheet 3. Time Course of the Changes in Heart Rate (Scene 1-3)*

Columns	Explanation	Notes / Meaning of codes
Time (s)	Time course before and after the event in each Scene	Relative HR from the point of 0 s

*Infants whose HR data had noises or defects were excluded.

Sheet 4. Time Course of the Changes in Heart Rate (Leaving the room)*

Columns	Explanation	Notes / Meaning of codes
Time (s)	Time course before and after the experimenter left the room	Relative HR from the point of 0 s

*Infants whose HR data had noises or defects were excluded.

Sheet 4. Time Course of the Changes in Heart Rate (Stethoscope)*

Column	Explanation	Notes / Meaning of codes
Time (s)	Time course before and after before and after gazing at the stethoscope	Relative HR from the point of 0 s

*Infants whose HR data had noises or defects were excluded.

Sheet 5. Infants' Gage (Scene 1-3)*

Column	Explanation	Notes / Meaning of codes
Time (s)	Time course before and after the event in each Scene	0 = Infant did not gaze at the experimenter<br>1 = Infant gazed at the experimenter

*The infants whose gazes could not be coded were excluded.

Sheet 6. Looking time (Scene 1-3)*

Column	Explanation	Notes / Meaning of codes
Time bin (s)	The proportion of total viewing time directed to the experimenter as a function of time bin	successive 1-s time bin

*The infants whose gazes could not be coded were excluded.

Sheet 7. Infants' Gage and Looking time (Leaving the room)*

Column	Explanation	Notes / Meaning of codes
Time (s)	Time course before and after the experimenter left the room	0 = Infant did not gaze at the experimenter<br>1 = Infant gazed at the experimenter
Time bin (s)	The proportion of total viewing time directed to the experimenter as a function of time bin	successive 1-s time bin

*The infants whose gazes could not be coded were excluded.

Sheet 1. Charasteristics

ID	age	sex	Gestational age	Birth Weight	family	sibling	nursery	shyness	follow	wo_mom	Consultation frequency	Injection	white coat	Experience of crying	breast_feed	Cry
NC1	311	1	40	3018	3	0	0	0	1	0	1.00	109	1	0	1	0
NC2	340	0	41	4470	4	1	0	1	1	1	0.50	90	0	0	1	0
NC3	272	0	41	3454	3	0	0	1	1	1	1.00	7	1	0	1	0
NC4	379	0	36	2284	4	1	0	1	1	0	1.00	0	1	0	0	0
NC5	413	0	41	3332	4	0	0	1	1	1	0.50	139	1	0	0	0
NC6	411	0	38	2870	4	1	0	1	1	1	0.50	134	1	0	1	0
NC7	398	0	41	3655	4	1	1	1	1	1	1.00	136	1	1	1	0
NC8	468	1	38	2920	3	0	0	1	1	1	0.50	86	1	0	0	0
NC9	617	1	38	2960	3	0	1	1	1	0	1.00	29	0	1	0	0
NC10	554	1	39	2998	5	0	0	1	1	1	2.00	89	0	0	0	0
NC11	459	1	37	2846	3	0	0	0	1	1	0.75	13	1	0	0	0
NC12	230	1	39	3798	4	1	0	1	1	1	2.00	14	1	1	0	0
NC13	242	1	41	3655	5	1	0	1	1	1	1.50	88	1	0	1	0
NC14	417	0	40	3090	3	0	0	1	0	0	1.00	6	1	0	1	0
NC15	498	1	37	1860	6	1	1	1	1	1	3.00	93	1	0	1	0
NC16	589	0	40	2632	5	1	1	1	1	1	2.00	63	1	0	1	0
NC17	402	0	38	3078	5	0	1	1	1	0	1.00	252	1	0	1	0
NC18	245	0	37	2030	4	1	0	1	1	1	1.00	5	0	0	1	0
NC19	487	0	37	3280	4	1	0	1	1	1	0.50	7	0	0	1	0
NC20	407	0	38	2980	3	0	0	1	1	1	3.50	30	0	0	1	0
NC21	263	0	39	3224	4	0	0	1	1	1	1.00	46	1	0	1	0
NC22	246	0	38	3592	3	0	0	0	0	0	1.50	77	1	0	1	0
NC23	249	1	40	3120	3	0	0	1	1	1	2.00	20	1	0	1	0
NC24	493	0	39	2922	4	0	1	1	1	0	1.00	13	1	0	0	0
NC25	367	1	38	2896	4	1	0	1	1	1	1.00	111	0	1	0	0
NC26	249	0	40	3362	3	0	0	1	1	1	4.00	0	1	1	1	0
NC27	244	1	40	3495	6	1	0	0	0	1	1.00	77	1	0	0	0
NC28	442	0	39	3175	4	1	0	1	1	1	0.33	61	1	0	0	0
NC29	176	1	41	3374	4	0	0	1	1	1	1.50	11	1	0	1	0
NC30	263	1	39	3220	3	0	1	1	1	1	1.50	8	1	0	1	0
NC31	416	1	39	3090	3	0	0	1	1	1	1.00	9	0	0	0	0
NC32	197	0	38	3164	4	1	0	1	1	1	1.00	12	0	0	1	0
NC33	179	0	38	2740	5	1	0	0	1	1	2.00	14	0	0	1	0
C1	340	0	37	2638	3	0	0	0	1	1	2.00	137	1	1	1	1
C2	392	1	39	2700	3	0	0	1	1	0	1.00	1	1	1	0	1
C3	323	0	39	2660	4	1	0	1	1	1	0.40	130	0	0	1	1
C4	300	0	39	3004	3	0	1	1	1	1	1.00	4	0	0	1	1
C5	242	1	39	2480	3	0	0	0	1	1	1.00	15	1	0	1	1
C6	254	0	39	2866	4	1	1	1	1	1	2.00	20	1	1	1	1
C7	542	0	37	2960	4	1	0	1	1	1	2.50	53	1	1	0	1
C8	372	1	40	3570	4	1	0	1	1	0	1.00	134	1	0	1	1
C9	469	0	40	3176	3	0	1	0	1	1	0.25	2	0	1	0	1
C10	368	0	39	3124	5	1	0	1	1	0	2.50	1	1	0	1	1
C11	425	0	37	2480	4	1	0	1	1	1	0.50	221	1	1	0	1
C12	245	0	37	2214	4	1	0	1	1	1	1.00	5	0	0	1	1
C13	453	0	40	3116	3	0	0	1	1	0	1.00	8	1	1	1	1
C14	512	1	38	2564	4	1	0	1	1	0	2.00	91	1	1	0	1
C15	496	0	37	2952	4	1	1	1	1	1	1.50	1	1	0	0	1
C16	479	1	38	3330	4	1	0	1	1	1	1.00	37	0	1	1	1
C17	368	1	39	3575	3	0	0	1	1	1	0.50	105	1	1	0	1
C18	372	1	39	2882	3	0	0	0	0	1	1.00	6	1	1	0	1
C19	436	1	39	2540	3	0	0	1	1	1	1.00	60	0	1	0	1
C20	187	1	39	3138	5	1	0	0	0	0	1.00	6	1	0	1	1
C21	419	1	38	2864	4	1	0	0	1	0	1.50	6	1	0	1	1
C22	377	1	41	3398	3	0	0	1	1	1	0.40	0	1	0	1	1
C23	455	0	38	2565	5	1	0	0	1	1	1.00	14	1	0	0	1
C24	376	0	37	2256	4	1	1	1	1	1	1.00	10	1	1	0	1
C25	375	1	41	2940	5	0	0	1	1	1	2.50	7	0	1	0	1
C26	420	1	40	2570	4	1	0	0	1	1	1.00	35	0	0	0	1
C27	529	0	38	3088	5	1	0	1	1	1	1.00	75	1	1	1	1
C28	547	1	37	3000	4	1	0	0	1	1	0.75	30	0	1	1	1

Sheet 2. Infants Who Cried in Each Scene.

ID	Time (s)															
	-5	-4	-3	-2	-1	0	1	2	3	4	5	6	7	8	9	10
First contact (Scene1)																
C1																
C2																
C3	1	1	1	1	1	1	1	1	1	1						
C4																
C5																
C6																
C7																
C8	1	1	1	1	1	1	1	1								
C9																
C10	1		1	1	1											
C11	1	1	1	1	1	1	1	1						1	1	1
C12																
C13																
C14														1	1	1
C15		1	1	1	1											
C16																
C17																
C18																
C19																
C20																
C21	1	1	1	1	1	1	1									
C22	1	1	1	1		1	1	1								
C23	1	1	1	1	1	1						1	1	1	1	1
C24																
C25																
C26	1	1	1	1	1	1	1									
C27																
C28																
Interview (Scene2)																
C1																
C2																
C3	1	1	1	1	1	1	1	1	1					1	1	1
C4							1	1	1	1	1	1	1	1	1	1
C5																
C6																
C7																
C8																
C9																
C10																
C11				1	1						1	1	1	1		
C12																
C13																
C14	1						1	1	1	1	1	1	1	1	1	
C15																
C16																
C17			1	1	1	1	1									
C18																
C19							1	1	1	1	1	1				
C20																
C21																
C22											1	1	1	1	1	
C23	1	1	1	1	1	1	1	1	1	1	1	1	1	1	1	1
C24																
C25																
C26																
C27																
C28																
Auscultation (Scene3)																
C1						1	1	1								
C2					1	1										
C3	1	1	1	1	1	1	1	1	1	1	1	1	1	1	1	1
C4	1	1	1	1	1	1	1	1	1	1	1	1	1	1	1	1
C5								1	1	1	1	1	1	1	1	1
C6						1	1	1	1	1	1	1	1	1	1	1
C7																
C8																
C9	1	1	1	1	1	1	1	1	1	1	1	1	1	1	1	1
C10																
C11									1	1	1	1	1			
C12													1	1	1	1
C13					1	1	1	1	1	1	1	1	1	1	1	1
C14	1	1	1	1	1	1	1	1	1	1	1	1	1	1	1	1
C15																
C16										1	1	1	1	1	1	1
C17						1	1	1	1	1	1	1	1	1	1	1
C18							1	1	1	1	1	1	1	1	1	1
C19					1	1	1	1	1	1	1	1	1	1	1	1
C20																
C21																
C22													1	1	1	1
C23	1	1	1	1	1	1	1	1	1	1	1	1	1	1	1	1
C24														1	1	1
C25						1	1	1	1	1	1	1	1	1	1	1
C26																
C27	1	1	1	1	1	1	1	1	1	1	1	1	1	1	1	1
C28	1	1	1	1	1	1	1	1	1	1	1	1	1	1	1	
Leaving																
C1																
C2																
C3			1	1	1	1	1	1	1	1	1					
C4	1	1	1	1	1	1	1	1	1	1	1					
C5																
C6	1	1	1		1				1	1	1					
C7			1	1				1	1	1	1					
C8																
C9																
C10																
C11	1	1	1	1	1	1	1		1	1						
C12																
C13	1	1	1	1	1	1	1	1	1	1	1					
C14	1	1	1	1	1	1	1	1	1	1	1					
C15											1					
C16																
C17																
C18																
C19	1	1	1	1	1	1	1	1	1							
C20																
C21																
C22																
C23		1	1	1												
C24																
C25																
C26																
C27																
C28	1	1	1	1	1	1	1	1	1	1						
Stethoscope																
C1																
C2							1	1								
C4	1	1	1	1	1	1	1	1	1	1	1	1	1	1	1	1
C5											1	1	1	1	1	1
C6								1	1	1	1	1	1	1	1	1
C7																
C8																
C10																
C11										1	1	1	1			
C12											1	1	1	1		
C14	1	1	1	1	1	1	1	1	1	1	1	1	1	1	1	1
C15																
C16											1	1	1	1	1	1
C17	1	1	1	1	1	1	1	1	1	1	1	1	1	1	1	1
C18														1	1	1
C19								1	1	1	1	1	1	1	1	1
C20																
C21																
C22															1	1
C24									1	1	1	1	1	1	1	1
C25											1	1	1	1	1	1
C26																
C28									1	1	1	1	1	1	1	1
	-5	-4	-3	-2	-1	0	1	2	3	4	5	6	7	8	9	10

Sheet 3. Time Course of the Changes in Heart Rate (Scene 1-3)

First contact (Scene1)

Time (s)

No.	ID	-5	-4.8	-4.6	-4.4	-4.2	-4	-3.8	-3.6	…
1	NC1	6.95181	0.734513	0.734513	4.546542	4.546542	4.546542	3.764956	3.764956	[illegible]
2	NC3	-3.107389	-3.107389	-3.107389	-3.107389	-3.107389	-3.107389	-3.107389	-4.772837	[illegible]
3	NC4	-5.769607	-5.769607	-4.707222	-4.707222	-4.707222	-14.0984	-14.0984	-14.0984	[illegible]
4	NC5	-1.495107	-1.495107	4.441083	4.441083	2.573091	2.573091	2.573091	-2.718884	[illegible]
5	NC6	9.992241	7.040998	7.040998	5.901288	5.901288	8.136906	8.136906	7.040998	[illegible]
6	NC7	5.573548	5.573548	5.573548	0	0	2.055102	2.055102	1.799612	[illegible]
7	NC8	-3.639626	-3.639626	-9.87006	-9.87006	-9.87006	-13.15739	-13.15739	-13.15739	[illegible]
8	NC9	-2.787245	-2.787245	-0.315914	-0.315914	2.558249	2.558249	1.037792	1.037792	[illegible]
9	NC10	-7.150581	-6.195635	-6.195635	-6.195635	-3.184566	-3.184566	-3.184566	-3.02141	[illegible]
10	NC13	7.671426	7.671426	11.34122	8.383679	8.383679	8.145529	8.145529	7.592693	[illegible]
11	NC14	-5.880903	-5.880903	-9.098866	-9.098866	-9.098866	-4.967148	-4.967148	-8.213265	[illegible]
12	NC15	8.444712	9.347464	9.347464	4.224624	4.224624	4.224624	0.52175	0.52175	[illegible]
13	NC16	-12.7691	-12.7691	-11.63849	-11.63849	-11.63849	-19.72425	-19.72425	-19.72425	[illegible]
14	NC17	-1.306105	-0.589753	-0.589753	-0.589753	-4.444344	-4.444344	-1.962199	-1.962199	[illegible]
15	NC18	12.56677	13.55447	13.55447	10.90212	10.90212	11.77472	11.77472	11.77472	[illegible]
16	NC21	-5.550423	-5.550423	-4.307722	-4.307722	1.700564	1.700564	4.100924	4.100924	[illegible]
17	NC22	4.004063	4.004063	4.004063	5.227443	5.227443	2.042189	2.042189	2.042189	[illegible]
18	NC23	-6.407682	-6.407682	-9.004712	-9.004712	-3.864983	-3.864983	-2.718199	-2.718199	[illegible]
19	NC24	-4.792037	-4.792037	-4.792037	-4.792037	-9.858818	-9.858818	-9.858818	-16.84064	[illegible]
20	NC25	1.518237	1.518237	3.071565	3.071565	3.287574	3.287574	-2.907696	-2.907696	[illegible]
21	NC29	3.141693	1.094407	1.094407	1.094407	-2.287486	-2.287486	-1.472882	-1.472882	[illegible]
22	NC30	-6.842085	-6.842085	-1.790161	-1.790161	-6.842085	-6.842085	-8.469348	-8.469348	[illegible]
23	NC31	-16.06732	-16.06732	-15.89649	-15.89649	-15.89649	-17.03428	-17.03428	-12.47914	[illegible]
24	NC32	-8.206781	-5.930792	-5.930792	-6.69691	-6.69691	-5.524526	-5.524526	-6.415534	[illegible]
[illegible]	[illegible]	[illegible]	[illegible]	[illegible]	[illegible]	[illegible]	[illegible]	[illegible]	[illegible]	[illegible]

Interview (Scene2)

Time (s)

[illegible]	[illegible]

Auscultation (Scene 3)

Time (s)

[illegible]	[illegible]

Sheet 4. Time Course of the Changes in Heart Rate (Leaving the room)

Leaving		Time (s)									
No.	ID	-5	-4.8	-4.6	-4.4	-4.2	-4	-3.8	-3.6	-3.4	-3.2
1	NC1	4.620932	4.620932	2.594477	2.594477	2.594477	1.93461	1.93461	8.897623	8.897623	8.897623
2	NC2	-3.087993	-3.087993	-3.087993	3.839418	3.839418	3.839418	-14.46995	-14.46995	-14.46995	-1.832429
3	NC3	6.713867	6.713867	5.373621	5.373621	6.922293	6.922293	6.922293	4.864749	4.864749	2.243386
4	NC4	0.044073	3.308658	3.308658	3.308658	11.32465	11.32465	10.03545	10.03545	10.03545	1.337056
5	NC5	7.165123	7.165123	5.475982	5.475982	2.905185	2.905185	2.905185	3.074844	3.074844	5.805065
6	NC6	-5.158928	-5.158928	-3.365967	-3.365967	-6.711806	-6.711806	-6.64642	-6.64642	-4.891171	-4.891171
7	NC7	-8.109213	-3.33897	-3.33897	-1.705799	-1.705799	-3.371247	-3.371247	-6.555884	-6.555884	-6.555884
8	NC8	21.15356	17.19424	17.19424	18.75315	18.75315	17.47246	17.47246	17.47246	2.01742	2.01742
9	NC9	4.946751	-0.672403	-0.672403	-0.672403	0.308357	0.308357	0.308357	3.467125	3.467125	6.465162
10	NC10	-2.0396	-3.031438	-3.031438	-3.031438	4.274216	4.274216	-5.864549	-5.864549	-5.864549	3.164198
11	NC11	4.727536	4.727536	4.727536	4.964346	4.964346	4.964346	1.665686	1.665686	1.665686	1.665686
12	NC12	1.711691	1.711691	1.711691	7.25739	7.25739	3.987087	3.987087	3.255431	3.255431	3.255431
13	NC13	0.225104	0.225104	3.928713	3.928713	0.3218	0.3218	0.3218	0.096384	0.096384	2.716929
14	NC14	3.161936	7.93828	7.93828	7.93828	7.33483	7.33483	2.881527	2.881527	2.881527	4.088607
15	NC15	2.846853	2.846853	-1.217815	-1.217815	0.330538	0.330538	0.330538	4.268203	4.268203	-0.626534
16	NC16	-19.51989	-19.51989	-9.378855	-9.378855	-9.378855	-9.378855	-9.307355	-9.307355	-9.307355	-11.58278
17	NC17	0.980903	0.980903	6.228096	6.228096	6.228096	14.35696	14.35696	14.35696	15.44542	15.44542
18	NC18	1.6399	1.6399	4.528098	4.528098	3.889827	3.889827	4.17632	4.17632	9.475606	9.475606
19	NC21	3.895832	3.895832	8.356632	8.356632	6.185887	6.185887	6.185887	4.671512	4.671512	6.249714
20	NC22	-2.179057	-2.938258	-2.938258	-2.296425	-2.296425	-0.904104	-0.904104	-0.904104	-1.648319	-1.648319
21	NC23	1.169958	1.169958	1.835524	1.835524	1.835524	-1.702626	-1.702626	1.169958	1.169958	0.141561
22	NC25	26.4529	24.89957	24.89957	19.08076	19.08076	19.08076	11.19103	11.19103	10.13204	10.13204
23	NC27	13.35234	13.35234	13.35234	8.959117	8.959117	15.17972	15.17972	9.221742	9.221742	10.04551
24	NC28	3.1596	3.1596	3.1596	5.973927	5.973927	10.45745	10.45745	4.521568	4.521568	3.009959
25	NC29	2.189663	2.189663	0.603688	0.603688	2.738226	2.738226	-0.948624	-0.948624	-0.878779	-0.878779
26	NC30	15.77203	11.03625	11.03625	17.26908	17.26908	14.12304	14.12304	16.70418	16.70418	16.70418
27	NC31	8.059615	5.221311	5.221311	3.887707	3.887707	3.887707	3.887707	3.887707	3.887707	3.887707
28	NC32	2.530692	2.530692	3.121501	3.121501	1.79873	1.79873	1.254475	1.254475	0.714213	0.714213
29	NC33	-1.65879	-1.65879	-1.65879	-2.067327	-2.067327	-2.098653	-2.098653	-0.738605	-0.738605	0.064599
30	C1	11.20222	11.20222	13.80075	13.94226	13.94226	13.47148	13.47148	9.797997	9.797997	15.08297
31	C2	10.33674	10.33674	10.67881	12.58661	12.58661	12.58661	12.58661	12.85029	12.85029	11.62706
32	C3	-2.719134	-2.719134	3.185344	3.250118	3.250118	6.544035	3.250118	3.250118	3.185344	6.544035
33	C4	3.185344	3.185344	3.250118	3.185344	3.185344	0	0	0	0	0
34	C5	35.07458	26.08224	26.08224	12.20655	12.20655	25.35967	25.35967	3.628926	3.628926	-0.608179
35	C6	8.026695	3.306149	3.306149	1.043397	1.043397	1.043397	5.586736	5.586736	-1.115287	-1.115287
36	C7	-4.552973	-2.317121	-2.317121	-2.317121	-5.255079	-5.255079	0.791204	0.791204	-0.75392	-0.75392
37	C8	-1.331767	-2.610337	-2.610337	0.185018	0.185018	0.185018	0	0	0	0
38	C10	2.002721	2.002721	2.002721	4.647664	4.647664	15.01845	15.01845	15.01845	-1.28328	-1.28328
39	C11	-4.893549	2.271136	2.271136	-4.065825	-4.065825	-3.123142	-3.123142	-3.123142	-4.100504	-4.100504
40	C13	2.598831	2.598831	-0.212822	-0.212822	0.986334	0.986334	1.941236	1.941236	1.159113	2.42295
41	C15	9.444022	8.024747	8.024747	8.024747	5.6038	5.6038	5.82912	5.82912	5.82912	6.662436
42	C16	0	0	3.042126	3.042126	3.042126	7.892211	7.892211	-7.080879	-7.080879	1.488797
43	C17	2.598941	2.598941	-3.27318	-3.27318	-3.27318	-1.804283	-1.804283	-1.804283	-14.44076	-14.44076
44	C18	15.23814	14.91214	14.91214	14.55831	14.55831	14.55831	9.142376	9.142376	6.752836	6.752836
45	C20	5.827834	11.05026	11.05026	11.05026	5.762479	5.762479	11.79274	11.79274	6.188381	6.188381
46	C21	-17.89009	-17.89009	-17.89009	-13.74928	-13.74928	-17.54017	-17.54017	-17.54017	-15.12896	-15.12896
47	C22	-0.472647	-6.958771	-6.958771	-6.958771	-4.545332	-4.545332	-4.545332	-1.810778	-1.810778	-1.810778
48	C23	3.978102	3.978102	4.28021	4.28021	-4.893365	-4.893365	-10.15122	-0.047974	-0.047974	-5.209574
49	C24	7.121352	7.121352	5.229288	5.229288	-0.277901	-0.277901	-0.277901	-3.696654	-3.696654	-1.990856
50	C25	7.386358	4.475028	4.475028	2.384706	2.384706	-0.266371	-0.266371	-0.266371	-3.212537	-3.212537
51	C26	2.010268	2.010268	2.010268	2.010268	3.063849	3.063849	3.063849	0	0	0
52	C27	24.17889	24.17889	16.39422	16.39422	16.39422	16.39422	14.93657	14.93657	14.93657	14.93657
53	C28	16.74275	16.74275	12.93218	12.93218	7.854726	7.854726	16.42766	16.42766	9.484709	9.484709

No.	ID	-3	-2.8	-2.6	-2.4	-2.2	-2	-1.8	-1.6	-1.4	-1.2	-1
1	NC1	1.282328	1.282328	5.3125	5.3125	5.3125	3.9375	3.9375	3.9375	3.262061	3.262061	3.9375
2	NC2	-1.832429	-1.832429	-16.50278	-16.50278	-16.50278	-16.51688	-16.51688	-16.51688	-3.585649	-3.585649	-3.585649
3	NC3	2.243386	0.888658	0.888658	1.853623	1.853623	-1.589651	-1.589651	3.260434	3.260434	3.260434	1.594986
4	NC4	1.337056	-1.948919	-1.948919	-1.948919	3.941512	3.941512	3.941512	4.676524	4.676524	-10.28905	-10.28905
5	NC5	5.805065	2.087868	2.087868	2.087868	1.116906	1.116906	2.543232	2.543232	2.543232	2.279179	2.279179
6	NC6	-1.772592	-1.772592	-1.527011	-1.527011	-0.714213	-0.714213	1.120944	1.120944	1.120944	1.120944	-0.678668
7	NC7	-6.555884	-6.555884	-1.705799	-1.705799	-1.672744	-1.672744	1.712955	1.712955	0	0	0
8	NC8	2.01742	9.727221	9.727221	13.4743	13.4743	13.4743	0.942128	0.942128	0.942128	8.297806	8.297806
9	NC9	6.465162	6.465162	2.838346	2.838346	2.838346	-1.376705	-1.376705	-2.524227	-2.524227	-2.524227	2.323181
10	NC10	3.164198	3.164198	-3.986383	-3.986383	-3.986383	-0.020368	-0.020368	2.096581	2.096581	2.096581	-7.571708
11	NC11	-17.71165	-17.71165	0.325296	0.325296	0.325296	-6.120758	-6.120758	-6.120758	-6.120758	-22.75534	-22.75534
12	NC12	4.534507	4.534507	4.178152	4.178152	6.544632	6.544632	6.544632	1.109849	1.109849	1.527909	1.527909
13	NC13	2.716929	2.085635	2.085635	0.225104	0.225104	3.590029	3.590029	1.198162	1.198162	0.128541	0.128541
14	NC14	4.088607	8.242144	8.242144	9.106267	9.106267	10.86974	10.86974	10.86974	4.89776	4.89776	-1.200991
15	NC15	-0.626534	1.915397	1.915397	2.566079	2.566079	-0.448123	-0.448123	-0.448123	1.454404	1.454404	2.379536
16	NC16	-11.58278	-11.58278	-1.804503	-1.804503	-1.804503	-2.435947	-2.435947	-2.435947	-0.893281	-0.893281	-0.893281
17	NC17	15.44542	8.139769	8.139769	8.139769	-8.395302	-8.395302	-8.395302	3.475908	3.475908	3.475908	2.584335
18	NC18	7.642289	7.642289	6.709983	6.709983	6.709983	7.978253	7.978253	8.214377	8.214377	8.349657	8.349657
19	NC21	6.249714	6.601817	6.601817	1.382474	1.382474	-1.211406	-1.211406	-1.211406	3.128896	3.128896	-0.406285
20	NC22	1.534255	1.534255	-2.705584	-2.705584	1.410232	1.410232	1.410232	-3.025299	-3.025299	-2.179057	-2.179057
21	NC23	0.141561	0.141561	1.835524	1.835524	-3.468591	-3.468591	-1.454224	-1.454224	-1.454224	-0.815034	-0.815034
22	NC25	10.13204	12.44219	12.44219	12.44219	7.609841	7.609841	11.19103	11.19103	11.19103	0	0
23	NC27	10.04551	10.04551	14.32299	14.32299	8.900898	8.900898	12.27277	12.27277	8.148683	8.148683	8.148683
24	NC28	3.009959	9.893404	9.893404	9.893404	7.171146	7.171146	5.414039	5.414039	-3.503025	-3.503025	0.891581
25	NC29	1.57284	1.57284	-1.781532	-1.781532	-1.470317	-1.470317	-2.091406	-2.091406	-2.091406	-2.673097	-2.673097
26	NC30	10.48258	10.48258	11.10576	11.10576	16.18075	16.18075	14.01441	14.01441	10.55156	10.55156	6.984721
27	NC31	-0.024782	-0.024782	-0.024782	0	0	0	0	0	2.55594	2.55594	-1.251159
28	NC32	-0.142009	-0.142009	0.249182	0.249182	-1.547055	-1.547055	-0.495827	-0.495827	0.535006	0.535006	-0.742479
29	NC33	0.064599	1.567109	1.567109	1.864879	1.864879	1.864879	1.633171	1.633171	0	0	-1.627263
30	C1	15.08297	13.2839	13.19027	13.19027	13.56542	13.56542	14.17864	14.17864	9.038003	9.038003	10.24836
31	C2	11.62706	9.361391	9.361391	8.231479	8.231479	4.30133	4.30133	0.186892	0.186892	-1.001216	-1.001216
32	C3	6.544035	3.250118	6.544035	6.544035	3.250118	6.544035	6.544035	3.250118	3.185344	3.185344	10.01917
33	C4	-0.062657	0	0	-3.144709	0	0	0	6.544035	6.544035	3.250118	3.185344
34	C5	-0.608179	-7.597861	-7.597861	-4.780708	-4.780708	-4.780708	-11.4733	-11.4733	-5.673969	-5.673969	-7.39645
35	C6	-3.300136	-3.300136	3.306149	3.306149	3.621664	3.621664	-1.115287	-3.300136	-3.300136	-1.115287	-1.115287
36	C7	-0.75392	-0.140279	-0.140279	-1.279229	-1.279229	0.5925	0.5925	0.5925	-2.127192	-2.127192	-0.531451
37	C8	0	-1.331767	-1.331767	0	0	1.333604	1.333604	1.333604	0	0	-1.331767
38	C10	1.311837	1.311837	11.49265	11.49265	11.49265	-0.046318	-0.046318	-0.046318	-7.58696	-7.58696	-3.919593
39	C11	-4.756255	-4.756255	-5.779598	-5.779598	-4.377339	-4.377339	-4.342792	-4.342792	-3.052823	-3.052823	-1.128133
40	C13	2.42295	2.554825	2.554825	2.510843	2.510843	3.217425	3.217425	2.730991	2.730991	2.203634	2.203634
41	C15	6.662436	0.296561	0.296561	0.296561	1.14892	1.14892	7.044971	7.044971	7.044971	5.82912	5.82912
42	C16	1.488797	1.488797	1.488797	1.488797	-8.386804	-8.386804	-0.02944	-0.02944	-2.937136	-2.937136	-2.937136
43	C17	-14.44076	-12.33819	-12.33819	-2.444697	-2.444697	-2.444697	-4.978851	-4.978851	-4.978851	-3.338078	-3.338078
44	C18	6.883235	6.883235	6.883235	4.350946	4.350946	1.606909	1.606909	1.606909	2.015482	2.015482	-0.810408
45	C20	8.662977	8.662977	9.34683	9.34683	12.25619	12.25619	5.177017	5.177017	3.57613	3.57613	3.57613
46	C21	-11.23156	-11.23156	-8.526765	-8.526765	-8.526765	-5.21913	-5.21913	-5.162072	-5.162072	-5.276138	-5.276138
47	C22	-4.719107	-4.719107	2.966698	2.966698	2.966698	1.132293	1.132293	1.132293	-5.868329	-5.868329	-5.868329
48	C23	-5.209574	-2.691886	-2.691886	0	0	-2.691886	5.550387	5.550387	2.681005	2.681005	2.730535
49	C24	-1.990856	1.547805	1.547805	3.347416	3.347416	5.191928	5.191928	3.347416	3.347416	3.347416	3.347416
50	C25	0.11873	0.11873	-0.825231	-0.825231	0.207915	0.207915	0.207915	-0.854514	-0.854514	-3.578686	-3.578686
51	C26	4.100745	4.100745	4.100745	-2.874823	-2.874823	4.253261	4.253261	4.253261	4.253261	-5.608931	-5.608931
52	C27	14.93657	13.50958	13.50958	0.708848	0.708848	5.301779	5.301779	5.301779	2.026067	2.026067	2.657364
53	C28	9.484709	6.157836	6.157836	11.15175	11.15175	12.93218	12.93218	9.448885	9.448885	4.56711	4.56711

No.	ID	-0.8	-0.6	-0.4	-0.2	0	0.2	0.4	0.6	0.8	1
1	NC1	3.9375	3.9375	3.9375	3.9375	0	0	0	-2.479167	-2.479167	-2.479167
2	NC2	-3.585649	-24.98016	-24.98016	-24.98016	0	0	0	0	-29.65547	-29.65547
3	NC3	1.594986	-1.589651	-1.589651	0	0	-1.187945	-1.187945	-0.408725	-0.408725	-0.408725
4	NC4	-10.28905	-7.37983	-7.37983	-7.37983	0	0	0	-4.852606	-4.852606	-14.00544
5	NC5	2.279179	-0.023045	-0.023045	0	0	0	-0.960194	-0.960194	-2.282855	-2.282855
6	NC6	-0.678668	-0.678668	-3.912006	-3.912006	0	0	-9.183561	-9.183561	-10.43346	-10.43346
7	NC7	0	0.23736	0.23736	0	0	0	-1.705799	-1.705799	-1.705799	-1.705799
8	NC8	7.499819	7.499819	7.499819	0	0	0	4.92542	4.92542	9.727221	9.727221
9	NC9	2.323181	2.323181	5.193341	5.193341	0	0	0	-6.102237	-6.102237	-6.102237
10	NC10	-7.571708	-7.571708	0	0	0	-5.882814	-5.882814	-5.882814	-4.943111	-4.943111
11	NC11	-22.75534	-1.24598	-1.24598	-1.24598	0	0	0	0	-29.1738	-29.1738
12	NC12	4.699648	4.699648	4.699648	0	0	3.878163	3.878163	3.878163	3.525437	3.525437
13	NC13	0.128541	1.230832	1.230832	0	0	-0.702747	-0.702747	3.286586	3.286586	-2.830574
14	NC14	-1.200991	-1.200991	1.597871	1.597871	0	0	0	-6.452812	-6.452812	-5.111368
15	NC15	2.379536	-1.978694	-1.978694	0	0	0	-2.500372	-2.500372	-3.189568	-3.189568
16	NC16	6.547272	6.547272	6.547272	0	0	0	-5.009586	-5.009586	-5.009586	0
17	NC17	2.584335	2.584335	0	0	0	2.584335	2.584335	2.584335	2.584335	2.584335
18	NC18	6.643859	6.643859	2.818144	2.818144	0	0	3.541276	3.541276	3.541276	3.257404
19	NC21	-0.406285	-2.147756	-2.147756	0	0	0	2.521544	2.521544	-3.596345	-3.596345
20	NC22	0.517761	0.517761	0	0	0	-8.819015	-8.819015	-3.832186	-3.832186	-6.27748
21	NC23	-0.169469	-0.169469	0	0	0	-3.307614	-3.307614	-1.260357	-1.260357	-9.101706
22	NC25	6.47782	6.47782	6.47782	0	0	0	-4.833529	-4.833529	-4.833529	4.235041
23	NC27	6.501141	6.501141	4.648649	4.648649	0	0	0	1.667331	1.667331	0.380805
24	NC28	0.891581	0.891581	2.711677	2.711677	0	0	-4.122332	-4.122332	-4.122332	-2.301249
25	NC29	-0.773886	-0.773886	-2.125754	-2.125754	0	0	-1.157753	-1.157753	-0.843832	-0.843832
26	NC30	6.984721	1.256169	1.256169	0	0	0	-3.266132	-3.266132	-5.401564	-5.401564
27	NC31	-1.251159	6.611026	6.611026	6.611026	0	0	9.685463	9.685463	6.611026	6.611026
28	NC32	-0.742479	2.01756	2.01756	0	0	2.751696	2.751696	-0.883049	-0.883049	0.786019
29	NC33	-1.627263	-3.247717	-3.247717	0	0	-0.032277	-0.032277	-0.032277	0.518478	0.518478
30	C1	11.8443	11.8443	5.895721	5.895721	0	0	3.742077	3.742077	-0.277485	-0.277485
31	C2	-2.82038	-2.82038	-1.148406	-1.148406	0	0	-1.734264	-1.734264	-6.328332	-6.328332
32	C3	3.250118	3.250118	6.544035	0	0	-6.189055	17.12095	17.12095	-0.18785	4.817582
33	C4	3.185344	0	3.185344	3.185344	0	3.250118	3.250118	-2.719134	-2.719134	3.250118
34	C5	-7.39645	-0.640041	-0.640041	-0.640041	0	0	4.514553	4.514553	4.480287	4.480287
35	C6	-2.842951	-2.842951	-0.344819	-0.344819	0	0	0.563499	0.563499	-0.430793	-0.430793
36	C7	-0.531451	1.938372	1.938372	1.938372	0	0	-5.280932	-5.280932	-3.604426	-3.604426
37	C8	-1.331767	2.750872	2.750872	2.750872	0	0	-0.026387	-0.026387	-0.026387	1.360565
38	C10	-3.919593	-3.919593	0	0	0	-4.629693	-4.629693	-16.53288	-16.53288	-16.53288
39	C11	-1.128133	-2.487812	-2.487812	0	0	1.220073	1.220073	1.294662	1.294662	-2.168058
40	C13	-5.593725	8.861659	8.861659	0	0	0.256137	0.256137	0	0	-0.127761
41	C15	1.708465	1.708465	1.708465	0	0	-8.050982	-8.050982	-8.050982	-3.808255	-3.808255
42	C16	-5.53127	-5.53127	-8.412646	-8.412646	0	0	0	-2.937136	-2.937136	-5.747274
43	C17	-3.682954	-3.682954	-3.682954	0	0	2.098448	2.098448	2.098448	3.491052	3.491052
44	C18	-0.810408	-0.810408	0.093331	0.093331	0	0	0	-3.747538	-3.747538	-3.747538
45	C20	-0.359379	-0.359379	-3.366683	-3.366683	0	0	-3.566167	-3.566167	-3.566167	-1.367249
46	C21	-5.276138	-6.040908	-6.040908	0	0	-3.690097	-3.690097	-6.545804	-6.545804	-6.545804
47	C22	0.47687	0.47687	0.47687	0	0	0	-18.50728	-18.50728	-18.50728	-19.60926
48	C23	2.730535	2.730535	2.681005	2.681005	0	0	-0.047974	-0.047974	-21.20516	-21.20516
49	C24	1.547805	1.547805	3.347416	3.347416	0	0	-3.696654	-3.696654	-1.990856	-1.990856
50	C25	-1.117487	-1.117487	0	0	0	1.348005	1.348005	-3.550593	-3.550593	-3.381785
51	C26	-1.934587	-1.934587	-1.934587	0	0	0	0	-8.182459	-8.182459	0.995399
52	C27	2.657364	2.657364	0.708848	0.708848	0	0	0	1.202578	1.202578	-0.363409
53	C28	-13.3294	-13.3294	14.71743	14.71743	0	0	6.157836	6.157836	6.157836	1.489306

No.	ID	1.2	1.4	1.6	1.8	2	2.2	2.4	2.6	2.8	3	3.2
1	NC1	1.93461	1.93461	1.93461	1.282328	1.282328	-1.869763	-1.869763	-1.869763	3.262061	3.262061	3.262061
2	NC2	-0.390988	-0.390988	-0.390988	-9.325542	-9.325542	-9.325542	-5.299921	-5.299921	-5.299921	2.859479	2.859479
3	NC3	-3.503324	-3.503324	-4.307078	-4.307078	-4.631777	-4.631777	0	0	-4.661216	-4.661216	-4.661216
4	NC4	-14.00544	-14.00544	-11.40915	-11.40915	-11.40915	-1.906328	-1.906328	-1.906328	1.246952	1.246952	-3.502543
5	NC5	-2.282855	0.67227	0.67227	0.976134	0.976134	0.976134	-1.52433	-1.52433	-0.687457	-0.687457	-0.687457
6	NC6	-9.873829	-9.873829	-6.679121	-6.679121	-6.679121	-21.55259	-21.55259	-27.0702	-27.0702	-27.0702	-21.73514
7	NC7	0	0	-1.705799	-1.705799	-1.705799	-1.705799	-3.33897	-3.33897	-3.371247	-3.371247	0
8	NC8	9.727221	-0.575817	-0.575817	-0.575817	1.534715	1.534715	1.534715	7.136905	7.136905	7.136905	-6.255852
9	NC9	-4.333794	-4.333794	-4.333794	1.897428	1.897428	0.680656	0.680656	0.680656	-0.367779	-0.367779	-0.367779
10	NC10	-1.049285	-1.049285	-1.049285	-15.16881	-15.16881	-15.16881	-2.0396	-2.0396	-2.0396	-6.787998	-6.787998
11	NC11	-29.1738	-29.1738	-22.0121	-22.0121	-22.0121	-2.889949	-2.889949	-2.889949	-2.565378	-2.565378	-2.565378
12	NC12	6.177051	6.177051	7.114216	7.114216	7.114216	-4.795272	-4.795272	7.400878	7.400878	6.516283	6.516283
13	NC13	-2.830574	-2.490824	-2.490824	-2.490824	1.656933	1.656933	0.354062	0.354062	-1.303963	-1.303963	2.085635
14	NC14	-5.111368	-5.111368	-5.444145	-5.444145	-5.444145	-10.68084	-10.68084	-7.315666	-7.315666	-7.315666	-6.082108
15	NC15	5.719365	5.719365	9.192512	9.192512	5.817193	5.817193	2.815598	2.815598	2.815598	-4.853549	-4.853549
16	NC16	0	0	-7.349149	-7.349149	-7.349149	-10.97964	-10.97964	-10.97964	-5.80219	-5.80219	-5.80219
17	NC17	2.584335	-4.645284	-4.645284	-4.645284	-2.425251	-2.425251	-2.425251	9.131607	9.131607	9.131607	9.131607
18	NC18	3.257404	1.82459	1.82459	3.131614	3.131614	0.784609	0.784609	3.446521	3.446521	3.446521	5.561698
19	NC21	-6.714308	-6.714308	-6.714308	-3.181806	-3.181806	0.23329	0.23329	-1.667072	-1.667072	-1.667072	-3.209525
20	NC22	-6.27748	9.551455	9.551455	9.551455	-0.242285	-0.242285	0.487194	0.487194	0.395578	0.395578	-2.090894
21	NC23	-9.101706	-9.101706	-4.161467	-4.161467	-5.238825	-5.238825	-5.238825	-5.783544	-5.783544	-1.81272	-1.81272
22	NC25	4.235041	-3.893826	-3.893826	-3.893826	-7.537587	-7.537587	-7.537587	3.294124	3.294124	3.294124	0.019991
23	NC27	0.380805	0.380805	1.823516	1.823516	0.81524	0.81524	0.81524	-4.322525	-4.322525	-2.431327	-2.431327
24	NC28	-2.301249	-8.694127	-8.694127	5.135809	5.135809	5.135809	-3.638171	-3.638171	-1.048482	-1.048482	-3.367592
25	NC29	-1.608801	-1.608801	-2.673097	-2.673097	-0.738888	-0.738888	-2.673097	-2.673097	-3.755355	-3.755355	-3.755355
26	NC30	-7.494407	-7.494407	-7.494407	-7.862819	-7.862819	-12.24353	-12.24353	-12.24353	-12.60899	-12.60899	-10.88435
27	NC31	6.611026	2.581782	2.581782	6.611026	6.611026	6.611026	2.55594	2.55594	1.27737	1.27737	-0.024782
28	NC32	0.786019	-2.411547	-2.411547	-1.997886	-1.997886	-0.354506	-0.354506	-1.616598	-1.616598	-1.616598	-2.136038
29	NC33	0.942594	0.942594	3.508432	3.508432	1.139209	1.139209	0.551011	0.551011	1.534101	1.534101	-1.12086
30	C1	-2.461955	-2.461955	-2.269435	-2.269435	-1.455491	-1.455491	-7.983524	-7.983524	-5.366401	-5.366401	-1.727772
31	C2	-7.550515	-7.550515	-4.279202	-4.279202	-13.88593	-13.88593	-9.639921	-9.639921	-9.639921	-11.06312	-11.06312
32	C3	4.817582	4.357836	5.478071	5.478071	-18.02223	-18.02223	-1.862264	12.26184	39.08599	39.08599	-1.369166
33	C4	-0.062657	-0.062657	-3.144709	0	0	0	-3.144709	-3.144709	-6.130296	-6.130296	-6.189055
34	C5	2.254371	2.254371	4.036314	4.036314	6.883706	6.883706	4.002276	4.002276	6.742179	6.742179	6.038647
35	C6	-0.688164	-3.795878	-3.795878	-3.009506	-3.009506	-6.547235	-6.547235	-4.124644	-4.124644	-6.507472	-6.507472
36	C7	-3.604426	-2.614442	-2.614442	-6.433304	-6.433304	-6.433304	-1.361782	-1.361782	-9.813933	-9.813933	-6.000479
37	C8	1.360565	1.549609	1.549609	4.143743	4.143743	5.595904	5.595904	5.595904	8.569676	8.569676	8.569676
38	C10	-16.53288	-41.86537	-41.86537	-41.86537	-15.82582	-15.82582	-15.82582	-4.135713	-4.135713	3.139596	3.139596
39	C11	-2.168058	-8.633951	-8.633951	-7.154803	-7.154803	-7.154803	-11.00008	-11.00008	-12.24599	-12.24599	-16.69459
40	C13	-0.127761	-0.085197	-0.085197	-2.186006	-2.186006	-0.805723	-1.477955	-1.477955	-4.272689	-4.272689	-4.313052
41	C15	-3.808255	-0.947942	-0.947942	-4.674681	-4.674681	-4.674681	-5.092526	-5.092526	-5.092526	-4.653707	-4.653707
42	C16	-5.747274	-7.080879	-7.080879	-7.080879	-8.412646	-8.412646	-8.412646	-8.412646	-8.412646	-10.96859	-10.96859
43	C17	6.478046	6.478046	6.478046	5.641987	5.641987	-2.532472	-2.532472	-2.532472	-8.102701	-8.102701	-8.102701
44	C18	-6.165588	-6.165588	-5.491256	-5.491256	-5.491256	-3.856735	-3.856735	-3.856735	-6.228393	-6.228393	-1.881358
45	C20	-1.367249	-10.59332	-10.59332	-3.765044	-3.765044	-3.765044	0.030035	0.030035	-5.057036	-5.057036	-3.195211
46	C21	-6.265792	-6.265792	-7.074512	-7.074512	-9.893664	-9.893664	-9.893664	-10.52682	-10.52682	-14.5415	-14.5415
47	C22	-19.60926	-19.60926	-5.647375	-5.647375	-5.647375	-5.647375	-12.12027	-12.12027	-12.12027	-25.71624	-25.71624
48	C23	-10.15122	-10.15122	-14.80458	-14.80458	-18.43546	-18.43546	-15.08393	-15.08393	-17.8614	-17.8614	-19.00521
49	C24	3.347416	3.347416	-6.988615	-6.988615	-6.988615	-3.696654	-3.696654	-5.329826	-5.329826	-0.277901	-0.277901
50	C25	-3.381785	-3.381785	-5.132679	-5.132679	-4.443554	-4.443554	-6.381611	-6.381611	-6.381611	1.833886	1.833886
51	C26	0.995399	0.995399	0.995399	-9.823722	-9.823722	-9.823722	-10.63733	-10.63733	-10.63733	-0.976484	-0.976484
52	C27	-0.363409	-0.363409	4.774371	4.774371	3.090368	3.090368	5.593746	5.593746	5.593746	1.276984	1.276984
53	C28	1.489306	0.851623	0.851623	-1.457647	-1.457647	-12.09075	-12.09075	-12.09075	-2.914669	-2.914669	-12.09075

No.	ID	3.4	3.6	3.8	4	4.2	4.4	4.6	4.8	5
1	NC1	3.262061	3.262061	0	0	0	1.282328	1.282328	4.620932	4.620932
2	NC2	2.859479	0.453147	0.453147	10.7141	10.7141	10.7141	2.838815	2.838815	5.87376
3	NC3	-4.631777	-4.631777	-2.931883	-2.931883	-1.589651	-1.589651	-1.620454	-1.620454	-1.620454
4	NC4	-3.502543	-3.502543	1.540302	1.540302	3.215475	3.215475	3.215475	2.544261	2.544261
5	NC5	1.778287	1.778287	1.778287	1.707073	1.707073	2.303136	2.303136	2.303136	0.976134
6	NC6	-21.73514	-21.29086	-21.29086	-21.29086	-31.23947	-31.23947	-34.58561	-34.58561	-34.58561
7	NC7	0	0	0	0	3.503115	3.503115	1.747634	1.747634	3.503115
8	NC8	-6.255852	-6.255852	1.534715	1.534715	5.604565	5.604565	5.604565	-0.907297	-0.907297
9	NC9	1.74917	1.74917	5.058708	5.058708	5.058708	6.213146	6.213146	6.213146	2.838346
10	NC10	-6.787998	4.274216	4.274216	1.028067	1.028067	1.028067	3.185756	3.185756	3.185756
11	NC11	-27.61281	-27.61281	-27.61281	-27.61281	-6.168605	-6.168605	0.543293	0.543293	0.543293
12	NC12	6.516283	4.89285	4.89285	9.740139	9.740139	9.206566	9.206566	13.09615	13.09615
13	NC13	2.085635	2.284382	2.284382	2.284382	2.284382	3.185725	3.185725	1.755633	1.755633
14	NC14	-6.082108	-6.082108	-6.669755	-6.669755	-8.501986	-8.501986	-8.501986	-7.508015	-7.508015
15	NC15	-4.937029	-4.937029	-2.874585	-2.874585	-2.874585	-5.32518	-5.32518	-6.58427	-6.58427
16	NC16	-7.349149	-7.349149	-7.349149	-7.349149	-9.550006	-9.550006	-9.550006	-5.694169	-5.694169
17	NC17	9.131607	5.288393	5.288393	5.288393	10.12192	10.12192	10.12192	10.14191	10.14191
18	NC18	5.561698	1.793773	1.793773	-1.600769	-1.600769	2.474976	2.474976	4.528098	4.528098
19	NC21	-3.209525	-0.896284	-0.896284	3.557311	3.557311	0.467404	0.467404	0.467404	1.531249
20	NC22	-2.090894	-2.179057	-2.179057	-2.179057	-10.41526	-10.41526	-7.425607	-7.425607	-7.425607
21	NC23	-3.815978	-3.815978	-3.815978	-3.334472	-3.334472	-1.121523	-1.121523	-0.507086	-0.507086
22	NC25	0.019991	0.019991	-3.893826	-3.893826	4.213483	4.213483	4.213483	-3.893826	-3.893826
23	NC27	-2.431327	-4.768569	-4.768569	0.329895	0.329895	6.75408	6.75408	6.75408	7.976309
24	NC28	-3.367592	-3.367592	-15.35621	-15.35621	-9.779909	-9.779909	-9.779909	-0.56884	-0.56884
25	NC29	-6.519856	-6.519856	-7.76709	-7.76709	-7.385714	-7.385714	-6.228822	-6.228822	-8.836321
26	NC30	-10.88435	-10.88435	-7.017544	-7.017544	-1.840894	-1.840894	0	0	1.498127
27	NC31	-0.024782	-0.024782	1.27737	1.27737	-0.024782	-0.024782	-0.024782	1.27737	1.27737
28	NC32	-2.136038	-4.61155	-4.61155	-3.604599	-3.604599	-3.163787	-3.163787	-2.170534	-2.170534
29	NC33	-1.12086	-1.12086	-0.866258	-0.866258	-2.969991	-2.969991	-1.65879	-1.65879	-2.90812
30	C1	-1.727772	0.398145	0.398145	-1.182237	-1.182237	1.930011	1.930011	2.707373	2.707373
31	C2	-10.09592	-10.09592	-13.94736	-13.94736	-13.57798	-13.57798	7.035619	7.035619	-23.48029
32	C3	-1.369166	3.444698	5.478071	5.478071	11.41509	1.134794	1.134794	3.965459	4.620287
33	C4	-9.137767	-9.137767	-6.130296	-6.189055	-6.189055	-3.144709	-3.144709	-9.08084	-6.189055
34	C5	6.038647	5.480697	5.480697	8.386516	8.386516	7.132041	7.132041	7.132041	8.967198
35	C6	-8.896134	-8.896134	-10.79965	-10.79965	-10.34717	-10.34717	-7.298865	-7.298865	-6.706083
36	C7	-6.000479	-6.000479	1.333608	1.333608	-6.229999	-6.229999	-6.229999	-0.781675	-0.781675
37	C8	8.569676	10.123	10.123	11.71266	11.71266	11.71266	7.051439	7.051439	5.595904
38	C10	3.139596	10.30377	10.30377	15.25444	15.25444	14.75396	14.75396	14.75396	3.876602
39	C11	-16.69459	-17.61232	-17.61232	-17.61232	-12.36934	-12.36934	-15.55854	-15.55854	-15.29358
40	C13	-4.313052	-5.593725	-5.593725	-2.928841	-2.928841	-1.686864	-1.686864	-1.686864	-1.686864
41	C15	6.687865	6.687865	10.35431	10.35431	10.35431	7.661979	7.661979	11.74462	11.74462
42	C16	-10.96859	-10.96859	-10.96859	-16.95537	-16.95537	-10.96859	-10.96859	-10.96859	-13.27873
43	C17	-1.066789	-1.066789	3.103649	3.103649	3.103649	3.734448	3.734448	6.63128	6.63128
44	C18	-1.881358	-1.881358	1.678808	1.678808	-0.902319	-0.902319	-0.902319	-6.353793	-6.353793
45	C20	-3.195211	1.518418	1.518418	1.518418	6.352881	6.352881	5.371626	5.371626	1.733743
46	C21	-14.5415	-15.49324	-15.49324	-17.82027	-17.82027	-17.82027	-19.36015	-19.36015	-21.47604
47	C22	-25.71624	-25.71624	-19.99976	-19.99976	-19.99976	-12.75761	-12.75761	-12.75761	-12.75761
48	C23	-16.34822	-16.34822	-16.34822	-36.0232	-36.0232	-24.19344	-24.19344	-26.34833	-26.34833
49	C24	-1.990856	-1.990856	-1.990856	-1.990856	-3.696654	-3.696654	-1.990856	-1.990856	3.347416
50	C25	1.620882	1.620882	1.408549	1.408549	-0.266371	-0.266371	2.384706	2.384706	2.384706
51	C26	-0.976484	-9.024923	-9.024923	-9.024923	0	0	0	3.213418	3.213418
52	C27	4.905809	4.905809	4.905809	8.981088	8.981088	10.22713	10.22713	0.73344	0.73344
53	C28	-12.09075	-13.30385	-13.30385	-13.30385	-17.95335	-17.95335	-15.66078	-15.66078	-15.66078

Sheet 4. Time Course of the Changes in Heart Rate (Stethoscope)

Stethoscope		Time (s)
No.	ID	[illegible]
[illegible]	[illegible]	[illegible]

Sheet 5. Infants' Gage (Scene 1-3)

First contact (Scene1) — Time (s)

No.	ID	-5	-4.8	-4.6	-4.4	-4.2	-4	-3.8	-3.6	-3.4	-3.2	-3	-2.8	-2.6	-2.4	-2.2	-2	-1.8	-1.6	-1.4	-1.2	-1	-0.8	-0.6	-0.4	-0.2	0	0.2	0.4	0.6	0.8	1	1.2	1.4	1.6	1.8	2	2.2	2.4	2.6	2.8	3	3.2	3.4	3.6	3.8	4	4.2	4.4	4.6	4.8	5	5.2	5.4	5.6	5.8	6	6.2	6.4	6.6	6.8	7	7.2	7.4	7.6	7.8	8	8.2	8.4	8.6	8.8	9	9.2	9.4	9.6	9.8	10
1	NC1	[illegible]
2	NC2	[illegible]
3	NC3	[illegible]
4	NC4	[illegible]
5	NC5	[illegible]
6	NC6	[illegible]
7	NC7	[illegible]
8	NC8	[illegible]
9	NC9	[illegible]
10	NC10	[illegible]
11	NC11	[illegible]
12	NC12	[illegible]
13	NC13	[illegible]
14	NC14	[illegible]
15	NC15	[illegible]
16	NC16	[illegible]
17	NC17	[illegible]
18	NC18	[illegible]
19	NC19	[illegible]
20	NC20	[illegible]
21	NC21	[illegible]
22	NC22	[illegible]
23	NC23	[illegible]
24	NC24	[illegible]
25	NC25	[illegible]
26	NC26	[illegible]
27	NC27	[illegible]
28	NC28	[illegible]
29	NC29	[illegible]
30	NC30	[illegible]
31	NC31	[illegible]
32	NC32	[illegible]
33	C2	[illegible]
34	C3	[illegible]
35	C4	[illegible]
36	C5	[illegible]
37	C6	[illegible]
38	C7	[illegible]
39	C8	[illegible]
40	C10	[illegible]
41	C11	[illegible]
42	C12	[illegible]
43	C13	[illegible]
44	C14	[illegible]
45	C15	[illegible]
46	C16	[illegible]
47	C17	[illegible]
48	C18	[illegible]
49	C19	[illegible]
50	C20	[illegible]
51	C21	[illegible]
52	C22	[illegible]
53	C23	[illegible]
54	C24	[illegible]
55	C25	[illegible]
56	C26	[illegible]
57	C28	[illegible]

Interview (Scene2) — Time (s)

No.	ID	-5	-4.8	-4.6	-4.4	-4.2	-4	-3.8	-3.6	-3.4	-3.2	-3	-2.8	-2.6	-2.4	-2.2	-2	-1.8	-1.6	-1.4	-1.2	-1	-0.8	-0.6	-0.4	-0.2	0	0.2	0.4	0.6	0.8	1	1.2	1.4	1.6	1.8	2	2.2	2.4	2.6	2.8	3	3.2	3.4	3.6	3.8	4	4.2	4.4	4.6	4.8	5	5.2	5.4	5.6	5.8	6	6.2	6.4	6.6	6.8	7	7.2	7.4	7.6	7.8	8	8.2	8.4	8.6	8.8	9	9.2	9.4	9.6	9.8	10
1	NC1	[illegible]
2	NC2	[illegible]
3	NC3	[illegible]
4	NC4	[illegible]
5	NC5	[illegible]
6	NC6	[illegible]
7	NC7	[illegible]
8	NC8	[illegible]
9	NC9	[illegible]
10	NC10	[illegible]
11	NC11	[illegible]
12	NC12	[illegible]
13	NC13	[illegible]
14	NC14	[illegible]
15	NC15	[illegible]
16	NC16	[illegible]
17	NC17	[illegible]
18	NC18	[illegible]
19	NC19	[illegible]
20	NC20	[illegible]
21	NC21	[illegible]
22	NC22	[illegible]
23	NC23	[illegible]
24	NC24	[illegible]
25	NC25	[illegible]
26	NC26	[illegible]
27	NC27	[illegible]
28	NC28	[illegible]
29	NC29	[illegible]
30	NC30	[illegible]
31	NC31	[illegible]
32	NC32	[illegible]
33	C2	[illegible]
34	C3	[illegible]
35	C4	[illegible]
36	C5	[illegible]
37	C6	[illegible]
38	C7	[illegible]
39	C8	[illegible]
40	C10	[illegible]
41	C11	[illegible]
42	C12	[illegible]
43	C13	[illegible]
44	C14	[illegible]
45	C15	[illegible]
46	C16	[illegible]
47	C17	[illegible]
48	C18	[illegible]
49	C19	[illegible]
50	C20	[illegible]
51	C21	[illegible]
52	C22	[illegible]
53	C23	[illegible]
54	C24	[illegible]
55	C25	[illegible]
56	C26	[illegible]
57	C28	[illegible]

Auscultation (Scene 3) — Time (s)

No.	ID	-5	-4.8	-4.6	-4.4	-4.2	-4	-3.8	-3.6	-3.4	-3.2	-3	-2.8	-2.6	-2.4	-2.2	-2	-1.8	-1.6	-1.4	-1.2	-1	-0.8	-0.6	-0.4	-0.2	0	0.2	0.4	0.6	0.8	1	1.2	1.4	1.6	1.8	2	2.2	2.4	2.6	2.8	3	3.2	3.4	3.6	3.8	4	4.2	4.4	4.6	4.8	5	5.2	5.4	5.6	5.8	6	6.2	6.4	6.6	6.8	7	7.2	7.4	7.6	7.8	8	8.2	8.4	8.6	8.8	9	9.2	9.4	9.6	9.8	10
1	NC1	[illegible]
2	NC2	[illegible]
3	NC3	[illegible]
4	NC4	[illegible]
5	NC5	[illegible]
6	NC6	[illegible]
7	NC7	[illegible]
8	NC8	[illegible]
9	NC9	[illegible]
10	NC10	[illegible]
11	NC11	[illegible]
12	NC12	[illegible]
13	NC13	[illegible]
14	NC14	[illegible]
15	NC15	[illegible]
16	NC16	[illegible]
17	NC17	[illegible]
18	NC18	[illegible]
19	NC19	[illegible]
20	NC20	[illegible]
21	NC21	[illegible]
22	NC22	[illegible]
23	NC23	[illegible]
24	NC24	[illegible]
25	NC25	[illegible]
26	NC26	[illegible]
27	NC27	[illegible]
28	NC28	[illegible]
29	NC29	[illegible]
30	NC30	[illegible]
31	NC31	[illegible]
32	NC32	[illegible]
33	C2	[illegible]
34	C3	[illegible]
35	C4	[illegible]
36	C5	[illegible]
37	C6	[illegible]
38	C7	[illegible]
39	C8	[illegible]
40	C10	[illegible]
41	C11	[illegible]
42	C12	[illegible]
43	C13	[illegible]
44	C14	[illegible]
45	C15	[illegible]
46	C16	[illegible]
47	C17	[illegible]
48	C18	[illegible]
49	C19	[illegible]
50	C20	[illegible]
51	C21	[illegible]
52	C22	[illegible]
53	C23	[illegible]
54	C24	[illegible]
55	C25	[illegible]
56	C26	[illegible]
57	C28	[illegible]

Sheet 6. Looking time (Scene 1-3)

First contact (Scene 1)		Time bin (s)														
No.	ID	-5	-4	-3	-2	-1	1	2	3	4	5	6	7	8	9	10
1	NC1	0.6	0	0	0	0.8	1	1	1	1	1	1	1	1	1	1
2	NC2	0	0	0	0	0	0	0.4	1	1	1	1	1	1	1	1
3	NC3	0	0	0.8	0.4	0	1	1	1	1	1	1	1	1	1	1
4	NC4	1	1	1	0.6	0.4	1	1	1	1	1	1	1	1	1	1
5	NC5	1	1	1	0	0	0	0	0.2	1	1	1	1	1	1	1
6	NC6	0	0	0	0	0	0	1	1	1	1	1	1	1	1	1
7	NC7	0.8	0.8	0	0	0	1	1	1	1	1	1	1	1	1	1
8	NC8	0.4	0	1	1	0.2	1	1	1	1	1	1	1	1	1	1
9	NC9	0	0	0	0	0	1	1	1	1	1	1	1	1	1	1
10	NC10	0	0.6	1	1	0.6	1	1	1	1	1	1	1	1	1	0.6
11	NC11	0	0	0	0	0	0	0	0	0.8	1	1	1	1	1	0.2
12	NC12	0	0	0	0	0	0	0	0	0	0.4	1	1	1	1	1
13	NC13	0	0	0	0	0	0	0	0	0.6	1	1	1	1	1	1
14	NC14	0	0	0	0	0	0	0.4	1	1	1	1	1	1	1	1
15	NC15	0	0	0	0	0	0	0	0	0	1	1	1	1	1	1
16	NC16	1	1	1	0.6	0	1	1	1	1	1	1	1	1	1	1
17	NC17	0	0.4	1	1	1	1	1	1	1	1	1	1	1	1	1
18	NC18	0	0	1	1	1	1	1	1	1	1	1	1	1	1	1
19	NC19	0	0	0	0	0	0	0	0	1	1	1	1	1	1	1
20	NC20	1	0	1	0	0	1	1	1	1	1	1	1	1	0	0
21	NC21	0	0	0	0	0	0	1	1	1	1	1	1	1	1	1
22	NC22	0	0	0	0	0	0	0	0	0.4	1	1	1	1	1	1
23	NC23	0	0	0.4	1	1	1	1	1	1	1	1	1	1	1	1
24	NC24	0	0	0	0	0	1	1	1	1	1	1	1	1	1	1
25	NC25	0	0	0	0	0	1	1	1	1	1	1	1	1	1	1
26	NC26	0	0.4	0.6	0	0.2	1	1	1	1	1	0.2	0.2	0.6	1	1
27	NC27	1	1	0.6	0	0	1	1	1	1	1	1	1	1	1	1
28	NC28	0	0	0	0	0	0.4	1	1	1	1	1	1	1	1	1
29	NC29	0	0	0	0	0	0	0	0	0	1	1	1	1	1	1
30	NC30	1	0.4	0.4	0	0.4	1	1	1	1	1	1	1	1	1	1
31	NC31	0	0	0	0	0	0	0.6	1	1	1	1	1	1	1	1
32	NC32	0.8	0.8	0	0	0	0	0	0.6	1	1	1	1	1	1	1
33	C2	0	0	0	0	0	1	1	1	1	1	1	1	1	1	1
34	C3	0	0	0	0	0	0	0	0	0	0.6	1	1	1	1	1
35	C4	0	0	0	0	0	0	0	0	1	1	1	1	1	1	1
36	C5	0	0	0	0	0	0.4	1	1	1	1	1	1	1	1	1
37	C6	0	0	0	0	0	0	0	0	0	0.6	1	1	1	1	1
38	C7	0	0	0	0	0	0	0	0	1	1	1	1	1	1	1
39	C8	1	0	0	0	0	0	0.2	1	1	1	1	1	1	1	1
40	C10	0	0	0	0	0.2	1	1	1	1	1	1	1	1	1	1
41	C11	0	0	0	0	0	1	1	1	1	1	1	1	1	1	1
42	C12	0	0	0	0	0	0	0	1	1	1	1	1	1	1	1
43	C13	0	0	0	0	0	0.4	1	1	1	1	1	1	1	1	1
44	C14	0	0	0	0	0	0	0	0.6	1	1	1	0.4	0.6	1	1
45	C15	0.8	0	0	0	0	1	1	1	1	1	1	1	1	1	1
46	C16	0	0	0	0	0	0.4	1	1	1	1	1	1	1	1	0.4
47	C17	0	0.8	1	0.6	0	0	0	0	1	1	1	1	1	1	1
48	C18	0	0.4	0.4	0.2	0	0	0.4	1	1	1	1	1	1	1	1
49	C19	0	0	0	0	0	0	1	1	1	1	1	1	1	1	1
50	C20	0	0	0	0	0	0	0	0	0.2	1	1	1	1	1	1
51	C21	0	0	0	0	0	0	0.4	1	1	1	1	1	1	1	1
52	C22	0	0	0	0	0	0	0	0	0.4	1	1	1	1	1	1
53	C23	0	0.4	1	1	1	1	1	1	1	1	1	1	1	1	1
54	C24	0	0	0	0	0	0	0.6	1	1	1	1	1	1	1	0.6
55	C25	0	0	0	0	0	0	1	1	1	1	1	1	1	0.6	0.4
56	C26	0	0	0	0	0	0	0	0	0	0.6	1	1	1	1	1
57	C28	1	1	1	1	1	1	1	1	1	1	1	1	0.8	0	0

Interview (Scene 2)		Time bin (s)														
No.	ID	-5	-4	-3	-2	-1	1	2	3	4	5	6	7	8	9	10
1	NC1	1	1	1	1	1	0.6	0.2	0.8	0	0.6	1	0.6	0	0	0.4
2	NC2	1	1	1	1	1	1	1	0.6	0	0.8	0.6	0	0	1	1
3	NC3	1	1	1	0.4	0	0.8	1	0.8	0	0.8	1	0	0.6	1	1
4	NC4	0.2	1	1	1	1	1	0.2	0.4	0.2	0	0	0	0.6	1	1
5	NC5	0	0.8	1	0.4	0.2	1	1	1	0	0	0	0	0	0	0.4
6	NC6	1	1	1	1	1	1	0.2	0	1	1	1	1	0.8	0	0
7	NC7	0	0.2	1	1	1	1	0	0	0	0	0.4	1	0.6	0	0
8	NC8	0	0	0	0	0	0	0	0.4	1	0.6	0	0	0	0	0.8
9	NC9	0	0.6	0	0	0	0.4	0	0	0	0	0.2	0.4	1	0.4	0
10	NC10	0	0	0	0	0	0.8	1	0.4	0	0	1	1	1	1	0
11	NC11	0	0	0	0	0	0.2	1	0.2	0	0	0	0	0	0	0
12	NC12	1	1	1	1	1	1	1	0.8	0	0	0	0.2	0.2	0	0
13	NC13	1	1	1	1	1	1	1	1	1	0.2	0	0.6	1	0.2	0.6
14	NC14	1	1	1	0.4	0	0.6	1	1	1	0.8	0	0	0	0.6	1
15	NC15	1	1	1	0.4	0.2	1	1	1	1	1	1	1	1	1	1
16	NC16	0.6	0	0	0	0	0	1	1	1	0.6	0	0	0	0	0
17	NC17	1	1	1	1	1	1	1	1	1	1	1	1	1	1	1
18	NC18	1	1	1	1	1	1	1	1	1	1	1	0.4	0	0	0.8
19	NC19	0	0	0	0	1	1	1	0	0	0	1	1	1	1	0
20	NC20	0	0	0	0	0	1	0	1	1	0	0	0	0	0	1
21	NC21	1	1	1	1	1	1	1	0.6	0	0	0.4	1	0.8	0	0
22	NC22	1	1	1	0.4	0	0.8	1	1	1	1	1	1	1	0.8	0
23	NC23	1	1	1	1	1	1	1	1	1	1	1	1	1	1	1
24	NC24	0.4	0.6	0.2	0	0.2	1	1	0.4	0	0	0	0.2	1	0.8	0
25	NC25	0	0	0	0	0	0.6	1	0.4	0	0	0	0	0	0.8	0
26	NC26	1	1	0.2	0	0	0	0	1	1	0.2	0	0.2	1	1	1
27	NC27	1	1	1	1	1	1	0.6	1	1	1	1	1	1	0.4	0
28	NC28	0.4	0	0	0	0.4	1	1	1	0.8	0	0	0.6	1	0.4	0
29	NC29	1	0.2	0	0.8	1	1	1	1	1	1	1	1	0.6	0	0
30	NC30	1	1	1	1	1	1	1	1	1	1	0.4	0	0	0.8	1
31	NC31	0.6	0	0.4	0.2	0.6	0	0	0	0.2	0.2	0.4	1	1	1	0.8
32	NC32	1	0	0	0	0	0	0	0	0	0	0	0	0	0.6	0.2
33	C2	0	0	0	0.8	1	1	0	0	0	0	0	0	0.2	0.2	0
34	C3	0	0	0	0	0	0	0	0	0	0	0	1	1	0.8	0
35	C4	0.2	0	0	1	1	1	0.4	0	0	0.4	0.4	0	0.6	0	0
36	C5	1	1	1	0.8	0.4	1	1	1	1	1	1	0	0.4	0	0.8
37	C6	1	1	1	1	1	1	1	0.2	0	1	1	1	1	1	1
38	C7	0	0	0	0	0	0.4	0.6	0	0	0	0	0	0.6	1	0.4
39	C8	1	1	0.2	0	0	0	0	1	0.2	0.4	1	1	1	1	0
40	C10	0.4	0	0	0	0	0.6	0.8	0	0	0	0.6	1	0.2	0	0
41	C11	1	1	1	1	0.6	0	0	0	0	0.6	1	1	1	0.2	0
42	C12	1	1	1	0.8	0	0.8	1	1	1	1	1	1	1	1	1
43	C13	1	1	1	1	1	1	1	1	1	1	1	1	1	1	1
44	C14	1	1	1	1	1	1	0.8	0	0	0	0	0	0.2	1	0
45	C15	0.4	0	0.6	1	1	1	1	0.4	0.6	1	1	1	1	1	1
46	C16	0.2	1	1	0.8	0	1	0.8	0	0.2	1	1	1	1	1	0
47	C17	1	1	0.6	0	0.2	1	1	1	1	1	1	1	0.4	0.6	0.6
48	C18	1	1	1	1	1	1	1	1	1	0.8	0	0.6	1	1	1
49	C19	1	1	1	0.2	0	0.8	1	0.6	0	0.4	1	0.2	0	0	0
50	C20	1	1	1	0.4	0	0.6	0.8	0	0	0.4	1	1	1	1	0.6
51	C21	1	1	1	1	1	0.6	0	0	0	0	0	0	0.4	1	1
52	C22	1	0	0	0.2	1	0.4	0	0.8	1	1	0	0	0	0	0.8
53	C23	1	1	1	1	1	0.8	0	0	0.8	1	1	1	1	1	0.8
54	C24	0.4	1	1	0.6	0	0.2	1	1	0.6	0	0	0	0	0.2	1
55	C25	1	1	0.8	0	0.4	1	0.4	0	0	0	0	0.2	1	1	0
56	C26	0.6	0	0.8	1	1	1	1	1	1	1	0.2	0	1	1	1
57	C28	0	0.4	1	1	0	0	0.4	0.4	0.2	0.6	0.6	0.8	0	0	0

Auscultation (Scene 3)		Time bin (s)														
No.	ID	-5	-4	-3	-2	-1	1	2	3	4	5	6	7	8	9	10
1	NC1	1	0.4	0	1	1	1	1	1	1	1	1	1	1	1	1
2	NC2	1	1	1	1	1	1	1	1	1	1	1	1	1	1	1
3	NC3	1	1	1	0.6	0.6	1	1	1	1	1	1	0	0	0	0
4	NC4	1	1	0.8	0	0	0	0	0	1	1	0.8	0	0	0	0
5	NC5	0	0.6	1	1	1	0	0	0	0	0	0	0	0	0	0
6	NC6	0	0.8	1	1	1	1	1	1	1	0	0	0.6	1	1	1
7	NC7	0	0.4	1	1	0	0	0.2	1	0.6	0	0	0	0	0	0
8	NC8	0.2	0	1	0.4	0	0	0	0	0	0	0	0	0	0	0
9	NC9	0.6	0.6	0.4	0.4	0.4	1	0	0	0.8	1	0.2	0	0	0	0
10	NC10	0	0	0	0.4	0.8	0	0	0	0	0	0	0	0	0	0
11	NC11	1	1	1	0.8	0	0	0	0	0	0	0	0	0	0	0
12	NC12	0	0	0.6	1	1	1	1	0	0.6	1	1	1	1	0.8	0
13	NC13	0.6	1	1	0.6	0.6	1	1	1	0.6	0	0	0	0.8	1	0.4
14	NC14	1	0.6	0	0	0	0	0	0.4	1	1	1	1	0.6	1	0.2
15	NC15	1	1	1	1	1	1	1	1	1	1	1	1	1	1	1
16	NC16	0	0	0.8	1	0.6	1	0.4	0	0	0	0	0	0	0	0
17	NC17	1	1	1	0.8	0	0	0	0.4	1	1	1	1	1	1	1
18	NC18	0	0	1	1	1	1	0	0.2	1	1	1	0.8	0	0	0.2
19	NC19	0	0	0	0	0	0	0	0	0	0	1	1	0	0	0
20	NC20	0	0	0	0	1	1	0	0	1	1	1	0	0	1	1
21	NC21	0.4	1	1	1	0.8	0	0.4	0	1	1	0.6	0	0	0	0.8
22	NC22	0.2	1	1	1	1	0.4	0	0	0	0	0	0	0	0	0
23	NC23	1	1	1	1	1	1	1	1	1	1	1	1	1	1	1
24	NC24	0.6	1	0.8	0	0	0	0.6	1	1	1	1	0.2	0	0	0
25	NC25	0	1	1	0.8	0	0	0	0.8	1	1	1	1	1	0.2	0.2
26	NC26	1	1	1	1	0	1	1	1	0	0	0	0	0	1	1
27	NC27	0	0.4	1	1	0.4	0.8	1	1	1	1	1	1	1	0.6	0.4
28	NC28	0.8	1	0.2	0	0	0	0	0	0.8	1	1	0.4	0	0	0
29	NC29	1	1	1	1	1	1	1	1	1	0.6	0	0	0	0	0
30	NC30	0.4	1	1	1	0	0	0	0	0.8	0.4	1	1	0.8	0	0.4
31	NC31	0.4	1	1	1	1	0	0	0.4	1	1	1	1	0.4	1	1
32	NC32	1	1	1	0.6	0	0	0	0	0	0	0	0	0	0	0
33	C2	1	1	1	0.4	0	0.2	1	1	0	0.2	1	1	0.4	0	0
34	C3	0	0	0	0	0	0	0	0	0	0	0	0	0	0	0
35	C4	0	0	0	0	0	0	0	0	0	0	0	0	0	0	0
36	C5	1	1	0.4	0	0	0.8	1	1	1	1	1	0.8	0	0	0.2
37	C6	1	1	1	1	0.2	0	0	0	0	0	0	0	0	0.2	0.2
38	C7	0	0.8	1	0.2	0	0	0.8	0.8	0	0	0	0	0.6	0	0
39	C8	1	1	1	1	0	0	0	0.8	1	0.6	0	0	0	0	0
40	C10	0	0	0	0	0	0	0	0	0	0	0	0	0	0	0
41	C11	1	0.4	0	0	0	0	0.4	0.6	0	0	0	0	0	0	0
42	C12	1	1	1	1	1	0.4	0	0	0	1	0.6	0	0	0	0.4
43	C13	1	1	1	1	1	0	0	0	0	0	0	0	0	0	0
44	C14	0	0	0	0	0	0.2	1	0	0	0	0	0	0	0	0
45	C15	1	1	1	1	0.6	0	0	0	0.4	1	1	0.6	0	0	0
46	C16	0.8	1	1	1	0.6	0.2	1	1	0.8	0.4	0.8	0.8	1	1	1
47	C17	0.6	1	1	1	1	0	0	0.8	0.4	0	0	0	0	0.6	1
48	C18	0	0.2	1	0.2	0	0	1	0.4	0	0	0	0	1	1	0
49	C19	1	1	0.6	0	0	0	0	0	0	0	0	0	0.8	0	0
50	C20	1	1	1	1	1	1	1	0.2	0	0	0	0	0.4	1	0
51	C21	0	0	0.4	1	1	0	0.4	0.6	0	0.2	1	1	1	0.6	0
52	C22	1	1	1	0.2	0.2	1	0.4	1	0.6	0	0	0	0	0	0
53	C23	1	1	1	1	1	0.8	0	0	0.2	1	1	1	1	1	1
54	C24	0	0.4	1	1	0.2	0.4	0	0.6	0.6	0	0	0	0.2	1	0.6
55	C25	0.4	1	0.6	0	0	0	0	0	0	0	0	0	0	0	0
56	C26	1	1	1	1	0.4	0	0.2	1	0.6	0.2	1	0	0	0	0
57	C28	0.8	0.4	0	0	0.8	0.4	0	0.2	1	0.6	1	1	1	1	1

Sheet 7. Infants' Gage and Looking time (Leaving the room)

Leaving		Time (s)																																																
No.	ID	-5	-4.8	-4.6	-4.4	-4.2	-4	-3.8	-3.6	-3.4	-3.2	-3	-2.8	-2.6	-2.4	-2.2	-2	-1.8	-1.6	-1.4	-1.2	-1	-0.8	-0.6	-0.4	-0.2	0	0.2	0.4	0.6	0.8	1	1.2	1.4	1.6	1.8	2	2.2	2.4	2.6	2.8	3	3.2	3.4	3.6	3.8	4	4.2	4.4	4.6	4.8	5
1	NC1	1	1	1	1	1	1	1	1	1	1	1	1	1	1	1	1	1	1	1	1	1	1	1	1	1	1	1	1	1	1	1	1	1	1	1	1	1	1	1	1	1	0	0	0	0	0	0	0	0	0	0
2	NC2	1	1	1	1	1	1	1	1	1	1	1	1	1	1	1	1	1	1	1	1	1	1	1	1	1	1	1	1	1	1	1	0	0	0	0	0	0	0	0	0	0	0	0	0	0	0	0	0	0	0	0
3	NC3	1	1	1	1	1	1	1	1	1	1	1	1	1	1	1	1	1	1	1	1	1	1	1	1	1	1	1	1	1	1	1	0	0	0	0	0	0	0	0	0	0	0	0	0	0	0	0	0	0	0	0
4	NC4	1	1	1	1	1	1	1	1	1	1	1	1	1	1	1	1	1	1	1	1	1	1	1	1	1	1	1	1	1	1	1	1	1	0	0	0	0	0	0	0	0	0	0	0	0	0	0	0	0	0	0
5	NC5	1	1	1	1	1	1	1	1	1	1	1	1	1	1	1	1	1	1	1	1	1	1	1	1	1	1	1	1	1	1	1	1	1	1	1	1	1	1	1	0	0	0	0	0	0	0	0	1	1	1	1
6	NC6	1	1	0	0	0	0	1	1	1	1	1	1	1	1	1	1	1	1	1	1	1	1	1	1	1	1	1	1	1	1	1	1	1	1	1	1	1	1	1	1	1	1	1	1	1	1	1	1	0	0	0
7	NC7	1	1	1	1	1	1	1	1	1	1	1	1	1	1	1	1	1	1	1	1	1	1	1	1	1	1	1	1	1	0	0	0	0	0	0	0	0	0	0	0	0	0	0	0	0	0	0	0	0	0	0
8	NC8	1	1	1	1	1	1	1	1	1	1	1	1	1	1	1	1	1	1	1	1	1	1	1	1	1	1	1	1	1	1	1	1	0	0	0	0	0	0	0	0	0	0	0	0	0	0	0	0	0	0	0
9	NC9	1	1	1	1	1	1	1	1	1	1	1	1	1	1	1	1	1	1	1	1	1	1	1	1	1	1	1	1	1	1	1	1	1	1	1	1	1	1	1	1	1	1	0	0	0	0	0	0	0	0	0
10	NC10	0	0	0	0	1	1	1	1	1	1	1	1	1	1	1	1	1	1	1	1	1	1	1	1	1	1	1	1	1	1	1	1	1	1	0	0	0	0	0	0	0	0	0	0	0	0	0	0	0	0	0
11	NC11	1	1	1	1	1	1	1	1	1	1	1	1	1	1	1	1	1	1	1	1	1	1	1	1	1	1	1	1	1	1	1	1	1	1	1	1	1	1	1	1	1	1	1	1	1	1	1	1	1	1	1
12	NC12	1	1	1	1	1	1	1	1	1	1	1	1	1	1	1	1	1	1	1	1	1	1	1	1	1	1	1	1	1	1	1	1	1	1	1	1	1	1	1	1	1	1	1	0	0	0	0	0	0	0	0
13	NC13	1	1	1	1	1	1	1	1	1	1	1	1	1	1	1	1	1	1	1	1	1	1	1	1	1	1	1	1	0	0	0	0	0	0	0	0	0	0	0	0	0	0	0	0	0	0	0	0	0	0	0
14	NC14	1	1	1	1	1	1	1	1	1	1	1	1	1	1	1	1	1	1	1	1	1	1	1	1	1	1	1	1	1	1	1	1	1	1	1	1	0	0	0	0	0	0	0	0	0	0	0	0	0	1	1
15	NC15	1	1	1	1	1	1	1	1	1	1	1	1	1	1	1	1	1	1	1	1	1	1	1	1	1	1	1	1	1	1	1	1	1	1	1	1	1	1	1	1	1	1	1	1	1	1	1	1	1	1	1
16	NC16	1	1	1	1	1	1	1	1	1	1	1	1	1	1	1	1	1	1	1	1	1	1	1	1	1	1	1	1	1	1	1	1	1	1	1	1	1	1	1	1	1	1	1	1	1	1	1	1	1	1	1
17	NC17	1	1	1	1	1	1	1	1	1	1	1	1	1	1	1	1	1	1	1	1	1	1	1	1	1	1	1	1	1	1	1	1	1	1	1	1	1	1	1	1	0	0	0	0	0	0	0	0	0	0	0
18	NC18	0	0	0	0	0	0	0	0	0	0	0	0	0	0	0	0	0	1	1	1	1	1	1	1	1	1	1	1	1	1	1	1	1	1	0	0	0	0	0	0	0	0	0	0	0	0	0	0	0	1	1
19	NC19	1	1	1	1	1	1	1	1	1	1	1	1	1	1	1	1	1	1	1	1	1	1	1	1	1	0	0	0	0	0	0	0	0	0	0	0	0	0	0	0	0	0	0	0	0	0	0	0	0	0	0
20	NC20	1	1	1	1	1	1	1	1	1	1	0	0	0	0	0	1	1	1	1	1	1	1	1	1	1	1	1	1	1	1	1	1	1	1	1	0	0	0	0	0	0	0	0	0	0	0	0	0	0	0	0
21	NC21	1	1	1	1	1	1	1	1	1	1	1	1	1	1	1	1	1	1	1	1	1	1	1	1	1	1	1	1	1	1	1	1	1	1	1	1	1	1	0	0	0	0	0	0	0	0	0	0	0	0	0
22	NC22	0	0	0	0	0	0	0	0	1	1	1	1	1	1	1	1	1	1	1	1	1	1	1	1	1	1	1	1	1	1	1	1	1	1	1	1	1	0	0	0	0	0	0	0	0	0	0	0	0	0	0
23	NC23	1	1	1	1	1	1	1	1	1	1	1	1	1	1	1	1	1	1	1	1	1	1	1	1	1	1	1	1	1	1	1	1	1	1	1	1	1	1	0	0	0	0	0	0	0	0	0	0	0	0	0
24	NC24	1	1	1	1	1	1	1	1	1	1	1	1	1	1	1	1	1	1	1	1	1	1	1	1	1	1	1	1	1	1	1	1	1	1	1	1	1	0	0	0	0	0	0	0	0	0	0	0	0	0	0
25	NC25	1	1	1	1	1	1	1	1	1	1	1	1	1	1	1	1	1	1	1	1	1	1	1	1	1	1	1	1	1	1	1	1	1	1	1	1	1	1	1	1	1	1	1	1	1	1	1	1	1	1	1
26	NC26	1	1	1	1	1	1	1	1	1	1	1	1	1	1	1	1	1	1	1	1	1	1	1	1	1	1	1	1	1	1	1	1	1	1	1	0	0	0	0	0	0	0	0	0	0	0	0	0	0	0	0
27	NC27	1	1	1	1	1	1	1	1	1	1	1	1	1	1	1	1	1	1	1	1	1	1	1	1	1	1	1	1	1	1	1	1	1	1	1	1	1	1	1	1	1	1	1	1	1	1	1	0	0	0	0
28	NC28	0	0	0	1	1	1	1	1	1	1	1	1	1	1	1	1	1	1	1	1	1	1	1	1	1	1	1	1	1	1	1	1	1	1	1	1	1	1	1	1	1	1	1	1	1	1	1	1	1	1	1
29	NC29	1	1	1	1	1	1	1	1	1	1	1	1	1	1	1	1	1	1	1	1	1	1	1	1	1	1	1	1	1	1	1	1	1	1	1	1	1	1	1	1	0	0	0	0	0	0	0	0	0	0	0
30	NC30	1	1	1	1	1	1	1	1	1	1	1	1	1	1	1	1	1	1	1	1	1	1	1	1	1	1	1	1	1	1	1	1	1	1	1	1	1	1	1	1	1	1	1	1	1	1	0	0	0	0	0
31	NC31	1	1	1	1	1	1	1	1	1	1	1	1	1	1	1	1	1	1	1	1	1	1	1	1	1	1	1	1	1	1	1	1	1	1	1	1	1	1	1	1	1	1	0	0	0	0	0	0	0	0	0
32	NC32	1	0	0	0	0	0	0	0	0	0	1	1	1	1	1	1	1	1	1	1	1	1	1	1	1	1	1	1	1	1	1	1	0	0	0	0	0	0	0	0	0	0	0	0	0	0	0	0	0	1	1
33	NC33	0	0	0	0	0	0	0	0	0	0	0	0	0	0	0	0	0	0	0	0	0	0	0	0	0	0	0	0	0	0	0	0	0	0	0	0	0	0	0	1	1	0	0	0	0	0	0	0	0	0	0
34	C1	1	1	1	1	1	1	1	1	1	1	1	1	1	1	1	1	1	1	1	1	1	1	1	1	1	1	1	1	1	1	1	1	1	1	1	1	1	1	1	1	1	1	1	1	1	1	1	1	1	1	1
35	C3	0	0	0	0	0	0	0	0	0	0	0	0	0	0	0	0	0	0	0	0	0	0	0	0	0	0	0	0	0	0	0	0	0	0	0	0	0	0	0	0	0	0	0	0	0	0	0	0	0	0	0
36	C4	1	1	1	1	1	1	1	1	1	1	1	1	1	1	1	1	1	1	1	1	1	1	1	1	1	1	1	1	1	1	1	1	1	1	1	1	1	1	1	1	1	1	1	1	0	0	0	0	0	0	0
37	C5	1	1	1	1	1	1	1	1	1	1	1	1	1	1	1	1	1	1	1	1	1	1	1	1	1	1	1	1	1	1	1	1	1	1	1	1	1	1	1	1	1	1	1	1	1	1	1	1	1	1	1
38	C6	0	0	0	0	0	0	0	0	0	0	0	0	0	0	0	0	0	0	0	0	0	0	0	0	0	0	0	1	1	1	1	1	1	1	1	1	0	0	0	0	0	0	0	0	0	0	0	0	0	0	0
39	C7	0	0	0	0	0	0	0	0	0	0	0	1	1	1	1	1	1	1	1	1	1	1	1	1	1	1	1	1	1	1	1	1	0	0	0	0	0	0	0	0	0	0	0	0	0	0	0	0	0	0	0
40	C8	1	1	1	1	1	1	1	1	1	1	1	1	1	1	1	1	1	1	1	1	1	1	1	1	1	1	1	1	1	1	0	0	0	0	0	0	0	0	0	0	0	1	1	1	1	1	1	1	1	1	0
41	C9	1	1	1	1	1	1	1	1	1	1	1	1	1	1	1	1	1	1	1	1	1	1	1	1	1	1	1	1	1	1	1	1	0	0	0	0	0	0	0	0	0	0	0	0	0	0	0	0	0	0	0
42	C10	1	1	1	1	1	1	1	1	1	1	1	1	1	1	1	1	1	1	1	1	1	1	1	1	1	1	1	1	1	1	1	1	1	1	1	1	1	1	0	0	0	0	0	0	0	0	0	0	0	0	0
43	C11	0	0	0	0	0	1	1	1	1	1	1	1	1	1	1	1	1	1	1	1	1	1	1	1	1	1	1	1	1	1	1	1	1	1	1	1	1	1	1	1	1	1	1	1	1	1	0	0	0	0	0
44	C12	1	1	1	1	1	1	1	1	1	1	1	0	0	0	0	0	0	0	0	0	0	0	0	0	0	0	0	0	0	0	0	0	0	0	0	0	0	0	0	0	0	1	1	1	1	1	1	1	1	1	1
45	C13	1	1	1	1	1	1	1	1	1	1	1	1	1	1	1	1	1	1	1	1	1	1	1	1	1	1	1	1	1	1	1	1	1	1	1	1	0	0	0	0	0	0	0	1	1	0	0	0	0	0	0
46	C15	1	1	1	1	1	1	1	1	1	1	1	1	1	1	1	1	1	1	1	1	1	1	1	1	1	1	1	1	1	1	1	1	1	1	1	1	1	1	1	1	0	0	0	0	0	0	0	0	0	0	0
47	C16	1	1	1	1	1	1	1	1	1	1	1	1	1	1	1	1	1	1	1	1	1	1	1	1	1	1	1	1	1	1	1	1	1	1	1	1	1	1	1	1	1	1	1	1	1	1	1	1	1	1	1
48	C17	1	1	1	1	1	1	1	1	1	1	1	1	1	1	1	1	1	1	1	1	1	1	1	1	1	1	1	1	1	1	1	1	1	1	1	1	1	1	1	1	1	1	1	1	1	1	1	0	0	0	0
49	C18	1	1	1	1	1	1	1	1	1	1	1	1	1	1	1	1	1	1	1	1	1	1	1	1	1	1	1	1	1	1	1	1	1	0	0	0	1	1	1	1	1	1	1	1	1	1	0	0	0	0	0
50	C19	1	1	1	1	1	1	1	1	1	1	1	1	1	1	1	1	1	1	1	1	1	1	1	1	1	1	1	1	1	1	1	1	1	1	1	1	1	1	1	1	1	1	1	1	1	1	1	1	1	1	1
51	C20	1	1	1	1	1	1	1	1	1	1	1	1	1	1	1	1	1	1	1	1	1	1	1	1	1	1	1	1	1	1	1	1	1	1	1	1	1	1	1	1	1	1	1	1	1	1	1	1	1	1	1
52	C21	1	1	1	1	1	1	1	1	1	1	1	1	1	1	1	1	1	1	1	1	1	1	1	1	1	1	1	1	1	1	1	1	1	1	1	1	1	1	1	1	1	1	1	1	1	1	1	1	1	1	1
53	C22	1	0	0	0	0	0	0	1	1	1	1	1	1	1	1	1	1	1	1	1	1	1	1	1	1	1	1	1	1	1	0	0	0	0	0	0	0	0	0	0	0	0	0	0	0	0	0	1	1	1	1
54	C23	1	1	1	1	1	1	1	1	1	1	1	1	1	1	1	1	1	1	1	1	1	1	1	1	1	1	1	1	1	1	1	1	1	0	0	0	0	0	0	0	0	0	1	1	1	1	1	1	1	1	1
55	C24	1	1	1	1	1	1	1	1	1	1	1	1	1	1	1	1	1	1	1	1	1	1	1	1	1	1	1	1	1	1	1	1	1	1	1	1	1	1	1	1	1	1	1	1	1	1	1	1	1	1	1
56	C25	1	1	1	1	1	1	1	1	1	1	1	1	1	1	1	1	1	1	1	1	1	1	1	1	1	1	1	1	1	1	1	1	1	1	1	0	0	0	0	0	0	0	0	0	0	0	0	0	0	0	0
57	C26	1	1	1	1	1	1	1	1	1	1	1	1	1	1	1	1	1	1	1	1	1	1	1	1	1	1	1	1	1	1	1	1	1	1	1	1	1	1	1	1	1	1	1	1	1	1	1	1	1	0	0
58	C28	0	0	0	0	1	1	1	1	1	1	1	1	1	1	1	1	1	1	1	1	1	1	1	1	1	1	1	1	1	1	1	1	1	1	1	1	1	1	1	1	1	1	1	1	1	1	1	1	0	0	0

Leaving		Time bin (s)									
No.	ID	-5	-4	-3	-2	-1	1	2	3	4	5
1	NC1	1	1	1	1	1	1	1	1	0.2	0
2	NC2	1	1	1	1	1	1	0.2	0	0	0
3	NC3	1	1	1	1	1	1	0.2	0	0	0
4	NC4	1	1	1	1	1	1	0.6	0	0	0
5	NC5	1	1	1	1	1	1	1	0.8	0	0.6
6	NC6	0.4	0.8	1	1	1	1	1	1	1	0.6
7	NC7	1	1	1	1	1	0.8	0	0	0	0
8	NC8	1	1	1	1	1	1	0.4	0	0	0
9	NC9	1	1	1	1	1	1	1	1	0.4	0
10	NC10	0.2	1	1	1	1	1	0.8	0	0	0
11	NC11	1	1	1	1	1	1	1	1	1	1
12	NC12	1	1	1	1	1	1	1	1	0.6	0
13	NC13	1	1	1	1	1	0.6	0	0	0	0
14	NC14	1	1	1	1	1	1	1	0.2	0	0.2
15	NC15	1	1	1	1	1	1	1	1	1	1
16	NC16	1	1	1	1	1	1	1	1	1	1
17	NC17	1	1	1	1	1	1	1	1	0	0
18	NC18	0	0	0	0.6	1	1	0.8	0	0	0.2
19	NC19	1	1	1	1	1	0	0	0	0	0
20	NC20	1	1	0	1	1	1	1	0	0	0
21	NC21	1	1	1	1	1	1	1	0.6	0	0
22	NC22	0	0.4	1	1	1	1	1	0.4	0	0
23	NC23	1	1	1	1	1	1	1	0.6	0	0
24	NC24	1	1	1	1	1	1	1	0.4	0	0
25	NC25	1	1	1	1	1	1	1	1	1	1
26	NC26	1	1	1	1	1	1	1	0	0	0
27	NC27	1	1	1	1	1	1	1	1	1	0.4
28	NC28	0.4	1	1	1	1	1	1	1	1	1
29	NC29	1	1	1	1	1	1	1	1	0	0
30	NC30	1	1	1	1	1	1	1	1	1	0.2
31	NC31	1	1	1	1	1	1	1	1	0.4	0
32	NC32	0.2	0	1	1	1	1	0.4	0	0	0.2
33	NC33	0	0	0	0	0	0	0	0.2	0.2	0
34	C1	1	1	1	1	1	1	1	1	1	1
35	C3	0	0	0	0	0	0	0	0	0	0
36	C4	1	1	1	1	1	1	1	1	0.8	0
37	C5	1	1	1	1	1	1	1	1	1	1
38	C6	0	0	0	0	0	0.6	1	0.2	0	0
39	C7	0	0	0.8	1	1	1	0.4	0	0	0
40	C8	1	1	1	1	1	1	0	0	0.8	1
41	C9	1	1	1	1	1	1	0.4	0	0	0
42	C10	1	1	1	1	1	1	1	0.6	0	0
43	C11	0	1	1	1	1	1	1	1	1	0.2
44	C12	1	1	0.2	0	0	0	0	0	0.8	1
45	C13	1	1	1	1	1	1	1	0.2	0.4	0
46	C15	1	1	1	1	1	1	1	1	0	0
47	C16	1	1	1	1	1	1	1	1	1	1
48	C17	1	1	1	1	1	1	1	1	1	0.4
49	C18	1	1	1	1	1	1	0.6	0.8	1	0.2
50	C19	1	1	1	1	1	1	1	1	1	1
51	C20	1	1	1	1	1	1	1	1	1	1
52	C21	1	1	1	1	1	1	1	1	1	1
53	C22	0.2	0.6	1	1	1	1	0	0	0	0.6
54	C23	1	1	1	1	1	1	0.6	0	0.6	1
55	C24	1	1	1	1	1	1	1	1	1	1
56	C25	1	1	1	1	1	1	1	0	0	0
57	C26	1	1	1	1	1	1	1	1	1	0.8
58	C28	0.2	1	1	1	1	1	1	1	1	0.6
