# Supplementary material for: The infant–doctor relationship: an examination of infants’ distress reactions in the presence of a doctor
Source: Sci Rep. 2024 Apr 4;14:7968. doi: 10.1038/s41598-024-58677-5 (PMC10994921; doi:10.1038/s41598-024-58677-5)
Supplement: Supplementary file 2 — Supplementary Figures. [file 41598_2024_58677_MOESM2_ESM.pdf]

Supplementary Figures	
**article title**	**The Infant-Doctor Relationship: An Examination of Infants' Distress Reactions in the Presence of a Doctor**
**journal name**	**Scientific Reports**
**author names**	**Motonobu Watanabe*, Masaharu Kato, Yoshi-Taka Matsuda, Kosuke Taniguchi, Shoji Itakura**
**affiliation and e-mail address of the corresponding author.**	***Center for Baby Science, Doshisha University, 4-1-1 Kizugawadai, Kizugawa-city, Kyoto 619-0226, Japan<br>E-mail: mowatana@mail.doshisha.ac.jp**

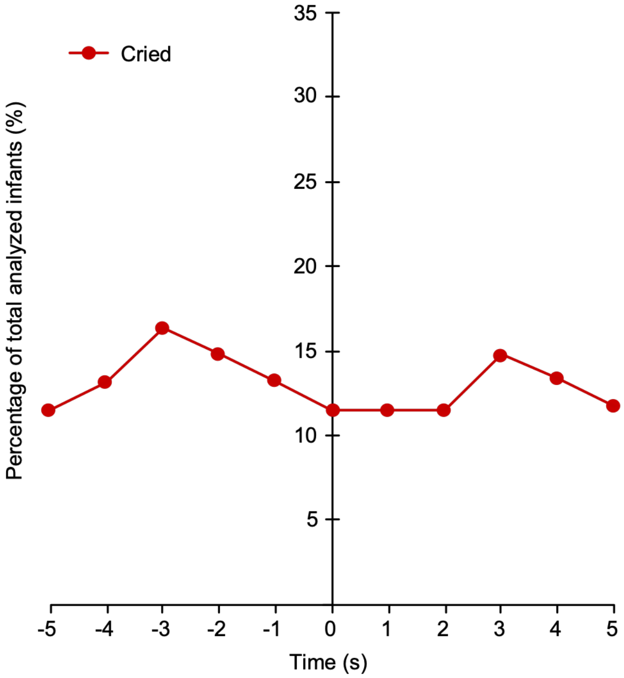

Supplementary Fig. S1 Changes in the percentages of infants who cried before and after the experimenter left the room

After examining the infant with a stethoscope, the experimenter left the room. We observed the infants' behaviors before and after the experimenter left the room to determine the subsequent effect on the infants. The moment at which the door was opened is the time point 0 s. We analyzed how many infants cried from 5 s before to 5 s after time point 0. Two coders who were unaware of the hypotheses of the study identified the infants' facial expressions, which were coded as negative or not. We defined vocalization with a negative face as crying.

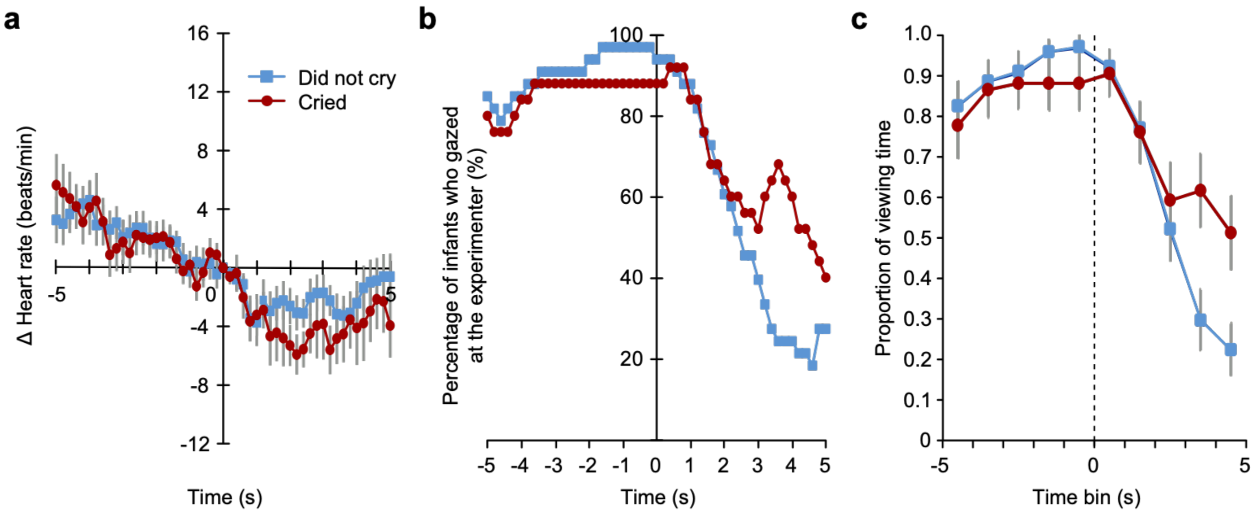

Supplementary Fig. S2 HR, gaze, and looking time before and after the experimenter left the room

(a) The event-related HR time courses in two groups of infants characterized by whether they cried are shown. Infants whose HR data had noise or defects were excluded. Twenty-four and 29 of the infants analyzed cried and did not cry, respectively. The results show the mean values for each group at each time point with the sample-by-sample intertrial SEM (vertical lines). **(b)** The percentages of infants in the two groups who gazed at the experimenter. Infants whose gazes could not be coded were excluded. In the scene without an experimenter, which occurred after he left the room (the time after the 0-s time point), we counted the number of infants who gazed in the direction of the entrance door. **(c)** The proportion of the total looking time directed at the experimenter for each successive 1-s time bin is shown. Time 0 is indicated here with a vertical dashed line. The symbols with error bars plotted in each time bin represent the average data and standard errors.
