# Supplementary material for: The infant–doctor relationship: an examination of infants’ distress reactions in the presence of a doctor
Source: Sci Rep. 2024 Apr 4;14:7968. doi: 10.1038/s41598-024-58677-5 (PMC10994921; doi:10.1038/s41598-024-58677-5)
Supplement: Supplementary file 3 — Supplementary Tables. [file 41598_2024_58677_MOESM3_ESM.pdf]

Supplementary Tables	
**article title**	**The Infant-Doctor Relationship: An Examination of Infants' Distress Reactions in the Presence of a Doctor**
**journal name**	**Scientific Reports**
**author names**	**Motonobu Watanabe*, Masaharu Kato, Yoshi-Taka Matsuda, Kosuke Taniguchi, Shoji Itakura**
**affiliation and e-mail address of the corresponding author.**	***Center for Baby Science, Doshisha University, 4-1-1 Kizugawadai, Kizugawa-city, Kyoto 619-0226, Japan<br>E-mail: mowatana@mail.doshisha.ac.jp**

Table S1. The effect of time and groups (infants who cried and infants who did not cry) on heart rate in each scene.

	Scene	Parameter	beta	Std.Error	t-value	p-value
The HR in each scene						
	First contact	intercept	1.36	1.20	1.13	0.259
		time	-0.40	0.10	-3.88	< 0.001
		groups	-0.30	1.25	-0.24	0.812
	Interview	intercept	2.53	1.42	1.77	0.077
		time	0.22	0.12	1.98	0.059
		groups	-3.73	1.52	-2.46	0.014
	Auscultation	intercept	-1.65	2.70	-0.61	0.541
		time	0.51	0.25	2.03	0.042
		groups	0.45	3.66	0.12	0.902
		time*groups	-0.82	0.34	-2.39	0.017
	Auscultation 2.5s after	intercept	2.21	1.92	1.16	0.248
		time	0.51	0.25	2.03	0.042
		groups	-5.70	2.60	-2.19	0.028
		time*groups	-0.82	0.34	-2.39	0.017
	Auscultation 6.25s after	intercept	4.15	2.14	1.94	0.053
		time	0.51	0.25	2.03	0.042
		groups	-8.78	2.90	-3.03	0.003
		time*groups	-0.82	0.34	-2.39	0.017
	Leaving	intercept	-2.35	1.36	-1.73	0.084
		time	-0.72	0.17	-4.16	< 0.001
		groups	3.10	1.43	2.17	0.030
The relative HR from the baseline						
	First contact	intercept	-0.63	0.96	-0.66	0.509
		time	-0.40	0.10	-3.88	< 0.001
		groups	-0.30	1.25	-0.24	0.812
	Interview	intercept	-2.05	1.26	-1.62	0.105
	Auscultation	intercept	5.25	3.30	1.59	0.111
		time	0.46	0.31	1.46	0.144
		groups	-9.00	4.47	-2.01	0.044
		time*groups	-0.76	0.43	-1.78	0.075

Note. Groups indicate two groups: infants who cried and infants who did not cry.

Table S2. The effect of time and groups (infants who cried and infants who did not cry) on looking time in each scene.

	Scene	Parameter	beta	Std.Error	t-value	p-value
Looking time						
	First contact	intercept	0.11	0.05	2.27	0.023
		time	0.06	0.00	13.89	< 0.001
		groups	0.09	0.05	1.83	0.067
	Interview	intercept	0.56	0.03	19.62	< 0.001
	Auscultation	intercept	0.53	0.06	8.99	< 0.001
		time	-0.02	0.01	-3.73	< 0.001
		groups	0.12	0.06	2.03	0.042
	Leaving	intercept	0.87	0.07	12.50	< 0.001
		time	-0.03	0.01	-2.64	0.009
		groups	0.11	0.09	1.14	0.253
		time*groups	-0.03	0.01	-2.25	0.025

Note. Groups indicate two groups: infants who cried and infants who did not cry.

Table S3. The effect of time and groups (infants who cried and infants who did not cry) on heart rate before and after the infants gazed at the stethoscope.

Time	Parameter	beta	Std.Error	t-value	p-value
Before and after gazing at the stethoscope	intercept	-0.70	1.99	-0.35	0.723
	time	0.43	0.20	2.16	0.031
	groups	0.98	2.60	0.38	0.707
	time*groups	-0.61	0.26	-2.30	0.021
2.5s after	intercept	2.55	1.29	1.98	0.048
	time	0.43	0.20	2.16	0.031
	groups	-3.57	1.69	-2.11	0.035
	time*groups	-0.61	0.26	-2.30	0.022
6.25s after	intercept	4.18	1.49	2.80	0.005
	time	0.43	0.20	2.16	0.031
	groups	-5.84	1.96	-2.99	0.003
	time*groups	-0.61	0.26	-2.30	0.021

Note. Groups indicate two groups: infants who cried and infants who did not cry.
